# Stilbenoids: A Natural Arsenal against Bacterial Pathogens

**DOI:** 10.3390/antibiotics9060336

**Published:** 2020-06-18

**Authors:** Luce Micaela Mattio, Giorgia Catinella, Sabrina Dallavalle, Andrea Pinto

**Affiliations:** Department of Food, Environmental and Nutritional Sciences (DeFENS), University of Milan, Via Celoria 2, 20133 Milan, Italy; luce.mattio@unimi.it (L.M.M.); giorgia.catinella@unimi.it (G.C.); andrea.pinto@unimi.it (A.P.)

**Keywords:** antibacterial activity, infectious diseases, stilbenoids, resveratrol derivatives, natural compounds

## Abstract

The escalating emergence of resistant bacterial strains is one of the most important threats to human health. With the increasing incidence of multi-drugs infections, there is an urgent need to restock our antibiotic arsenal. Natural products are an invaluable source of inspiration in drug design and development. One of the most widely distributed groups of natural products in the plant kingdom is represented by stilbenoids. Stilbenoids are synthesised by plants as means of protection against pathogens, whereby the potential antimicrobial activity of this class of natural compounds has attracted great interest in the last years. The purpose of this review is to provide an overview of recent achievements in the study of stilbenoids as antimicrobial agents, with particular emphasis on the sources, chemical structures, and the mechanism of action of the most promising natural compounds. Attention has been paid to the main structure modifications on the stilbenoid core that have expanded the antimicrobial activity with respect to the parent natural compounds, opening the possibility of their further development. The collected results highlight the therapeutic versatility of natural and synthetic resveratrol derivatives and provide a prospective insight into their potential development as antimicrobial agents.

## 1. Introduction

Natural products produced by living organisms, such as plants, fungi, bacteria, insects, sponges, and large animals as means of defence against pathogens and stress factors, have always been a source of inspiration for new drugs. Natural products constitute privileged structures in terms of chemical and biological space, which have been optimised after millennia of evolutionary pressure [1,2,3]. The antibiotic era started with Fleming’s discovery of the natural penicillins, which was firstly isolated from the fungus *Penicillium chrysogenum*, followed by aminoglycosides, cephalosporins, glycopeptides, macrolides, rifamycins, and tetracyclines [4]. To date, these natural product scaffolds are still valid lead compounds in the antibiotic research. Newman and Cragg [5] reported that in the last 40 years, 162 molecules were introduced as antibacterial agents: besides 4 biologics and the 32 vaccines, only 36 molecules are completely synthetic, mainly belonging to the quinolones family. Interestingly, quinolones derive from nalidixic acid, a by-product generated in the synthesis of derivatives of the natural compound quinine [4]. The remaining 90 antibacterial molecules, accounting for over 55% of the total, include 11 natural products, 78 semi-synthetic derivatives, and 1 synthetic compound containing a nature-inspired pharmacophore (bromodimoprin), confirming the key role that nature is still playing as source of new scaffolds for this class of drugs [5]. 

**Figure 1:**
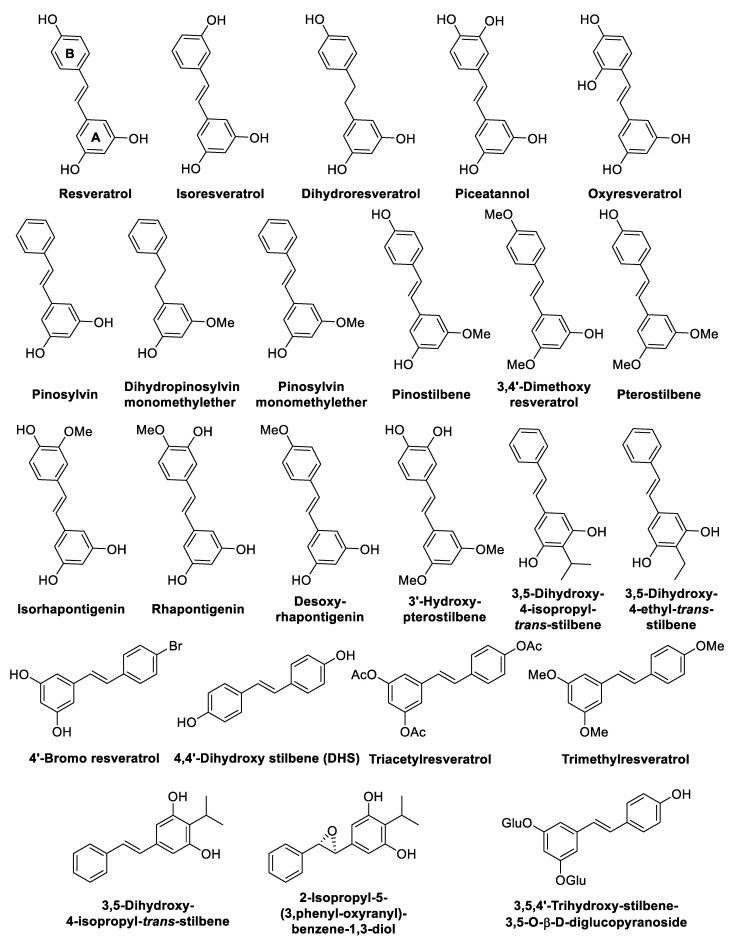
Structures of monomeric stilbenoids.

Stilbenoids represent an attractive class of plant polyphenols that are widely present in nature and largely studied in the last decades because of their different bioactivities such as anti-inflammatory, neuroprotective, anticancer, antimicrobial, and antidiabetic effects [6]. Stilbenoids are both woody constitutive metabolites and phytoalexins, which are substances produced by plants as means of protection against microbial infections and stress factors [7]. Stilbenoids biosynthesis occurs via the phenylpropanoid pathway: phenylalanine is converted into a cinnamate derivative, which, after activation into cinnamoyl–CoA by CoA–ligase, undergoes the enzymatic reaction of stilbene synthase to give the stilbenoid scaffold that can be further processed by several reactions such as glycosylation, methylation, prenylation, and oxidative couplings [6,8]. From a biochemical point of view, stilbenoids, including stilbenes, 2-aryl benzofurans, phenanthrenes, and related compounds, derive from the same biosynthesis pathway [9]. From a chemical point of view, stilbenoids share the stilbene backbone, consisting of two differently substituted aromatic rings, which is linked by an ethylene bridge, the *E*-configuration being the most common and stable in nature [9]. The aromatic rings differ in the number and position of functional groups, including hydroxy, methoxy, prenyl, geranyl, or farnesyl moieties. Moreover, stilbenoids can be classified as monomers or oligomers, which are isolated as aglycones or glycosides [6]. The most studied stilbenoid is resveratrol (Figure 1), which has been extensively investigated for its numerous potential health benefits [10]. However, resveratrol-derived monomers, dimers, and oligomers are endowed with multifaceted biological activities as well [6]. 

This review provides an overview of recent literature on the antibacterial potential of natural and nature-inspired stilbenoids, focusing on mechanisms of action, structure–activity relationship (SAR) studies, combination therapies, and potential applications in the medicinal and food chemistry field. 

## 2. Monomeric Stilbenoids 

### 2.1. Resveratrol

Resveratrol (3, 5, 4′-trihydroxystilbene) (Figure 1) is a natural phytoalexin that was first discovered in the roots of the white hellebore of *Veratrum grandiflorum* and then isolated from several plants and fruits, such as grapes, apples, berries, pistachios, and peanuts. Several experimental and preclinical studies attributed cardioprotective, cancer chemopreventive, anti-inflammatory, and antidiabetic effects to this molecule [10]. Resveratrol was tested on many different microorganisms for its antibacterial activity. 

In a recent review, Vestergaard et al. [11] described the multiple targets potentially involved in bacterial growth inhibition displayed by resveratrol. Resveratrol can reversibly bind to ATP synthase in the aerobic *E. coli* (IC_50,_ concentration of inhibitor where 50% of maximal inhibition was observed = 94 µM), and in *Mycobacterium smegmatis* (IC_50_ = 50 µM), thus reducing their cellular energy production and inhibiting their proliferation. Moreover, in *E. coli*, resveratrol was found to inhibit oxidative phosphorylation. In *E. Coli* mutans, lacking ATP synthase, resveratrol at a concentration of 228 µg/mL seemed to interfere with cell division, probably by the suppression of FtsZ, which is a key protein involved in septum formation during cell division. In addition, *E. coli* cells treated with 182 µg/mL resveratrol showed potassium leakage and propidium uptake, indicating membrane damage, whereas *S. aureus* species did not show membrane damage [11]. Resveratrol may also cleave the DNA, generating a Cu(II)–peroxide complex that binds to DNA to form a DNA–resveratrol–Cu(II) ternary complex. Through the reduction of Cu(II) to Cu(I), DNA cleavage occurs. In this mechanism, the 4-hydroxy group is fundamental in the reduction of copper, because of its pro-oxidant activity. As proof of concept, isoresveratrol (Figure 1), an analogue of resveratrol bearing an hydroxyl group in the *meta* position in place of the *para* position, is not able to reduce Cu(II) and to cause DNA cleavage. Moreover, the olefinic bridge plays a key role, providing the planarity to bind efficiently DNA and to stabilise the 4-oxy radical form. Conversely, dihydroresveratrol (Figure 1) showed a decreased ability of DNA cleavage [12].

In a study on the two Gram-negative *Arcobacter butzleri* and *Arcobacter cryaerophilus* [13], which are commonly found in contaminated food and water and are consequently associated with human and animal infections [14], resveratrol exerted bacteriostatic and bactericidal activity by different mechanisms of action, with minimum inhibitory concentration (MIC) values of 50–100 µg/mL. The DNA-specific fluorescent stain DRAQ5 revealed a reduced content of DNA in the treated bacterial cultures, highlighting the action of resveratrol on DNA synthesis. The decrease of metabolic activity and intracellular DNA content occurred before the membrane alteration, which was observed by SEM (scanning electron microscopy), suggesting that in this case, resveratrol did not target directly the membrane but acted on several cellular functions that eventually led to cell division impairment and death. Moreover, resveratrol showed a partial activity as efflux pump inhibitor (EPI) in the ethidium bromide (EtBr) accumulation assays. On the other hand, the presence of the effective EPI PAβN (l-phenylalanine-l-arginine-β-naphthylamide) enhanced the susceptibility to resveratrol with the reduction of MIC values by 16-fold and fourfold in *A. butzleri* and *A. cryaerophilus*, respectively. This result revealed the high contribution of efflux pumps to resistance to the polyphenol in *Arcobacter* spp [13]. Resveratrol antivirulence properties were demonstrated on several microorganisms [11,15]. Virulence is the ability of a pathogen to cause damage to a host through virulence factors (e.g., toxins) or the mechanism of infection (factors for adhesion, invasion, colonisation, and biofilm production). Therefore, the pathogen left without these abilities is not able to harm the host anymore [16,17]. Resveratrol inhibited biofilm formation in Gram-negative bacteria, such as *Fusobacterium nucleatum*, *V. cholerae*, *P. aeruginosa*, and *E. coli*, as well as in the Gram-positive *P. acnes* [11]. Through the downregulation of motility and flagella genes, resveratrol inhibited the motility in *P. mirabilis*, *E. coli*, and *Vibrio vulnificus*. In *V. vulnificus*, resveratrol reduced the expression of the toxin RtxA1, implicated in mice lethality, at 10–30 µM concentration, whereas in *V. cholerae*, resveratrol directly bound cholera toxin (CT) and prevented the toxin endocytosis into host cells at 300–400 µM concentration [11]. Moreover, in a recent study, resveratrol was found to inhibit at 32 µg/mL (concentration eightfold lower than MIC) the expression of alpha-hemolysin (Hla), which is a toxin secreted by several pathogenic *S. aureus* strains to create a pore in target cells, leading to membrane damages and cell death. In particular, RT-PCR assays revealed that the transcription levels of *hla* (encoding Hla) and *RNAIII*, the effector molecules of the accessory gene regulator (*agr*) locus, were reduced by 5.76 fold and 3.57 fold, respectively. In vitro studies on *S. aureus*-infected A459 cells verified that resveratrol at 32 µg/mL was able to alleviate the injury caused by *S. aureus*. Further, the in vivo experiments on mice with *S. aureus* pneumonia confirmed that resveratrol was able to reduce the mortality rate of infected mice by decreasing inflammatory reactions and bacterial burden in their lungs [18]. In *Yersinia enterolitica*, *Erwinia carotovora*, *E. coli*, and *Chromobacterium violaceum*, resveratrol (5–20 µg/mL) was also found to interfere with quorum sensing (QS) releasing factors [11]. These chemical signaling molecules (autoinducers) are produced by bacteria proportionally to cell density in order to regulate virulence factors production, biofilm formation, swarming motility, and sporulation. Therefore, QS helps bacteria invasion of the host when the number of bacterial cells is high, increasing the chances of successful infection, and thus the survival of bacterial pathogens [19]. Overall, resveratrol showed an antivirulence effect at concentration up to 64-fold below MIC values [11]. 

Resveratrol exhibited inhibitory activity on *Listeria monocytogenes* and *Listeria innocua* planktonic cells growth and biofilm formation at subinhibitory concentrations (sub-MIC) ranging from 50 to 100 µg/mL. These activities were observed in lettuce model medium and chicken juice, but not in milk, which was probably because of the reduced bioavailability due to the hydrophobic interactions between resveratrol and milk proteins or fats. However, these results encouraged the potential use of resveratrol as a food preservative for certain types of food matrix [20]. 

In a recent study [21], resveratrol was identified as a promising agent against the Gram-negative anaerobic *Porphyromonas gingivalis*, which is a keystone in periodontitis: a chronic inflammatory oral disease leading to bone and connective tissue destruction [22]. Resveratrol displayed bacteriostatic and bactericidal effects on *P. gingivalis* ATCC 33277, ATCC 53978, and CS02 strains with a MIC value of 156 µg/mL and minimum bactericidal concentration (MBC) value of 312 µg/mL, and on the CS01 strain with a MIC value of 78 µg/mL and MBC value of 156 µg/mL. Furthermore, at sub-MIC concentration, resveratrol inhibited biofilm formation by reducing the gene expression of virulence factors, including the genes type II and IV fimA (encoding fimbriae that allow bacteria to bind to host cells, and cysteine protease rgpA) and kgp genes (encoding gingipains, bacterial proteases involved in the cleavage of extracellular proteins, facilitating *P. gingivalis* biofilm growth) [21]. 

Resveratrol was used as pre-treatment at 1–100 µM concentration in *Helycobacter pylori*-infected cells, and it significantly inhibited IL-8 secretion by cells, suppressed reactive oxygen species (ROS) production, and remarkably blocked host cell morphological changes associated with cell dysregulation and pathogenesis [23]. Moreover, resveratrol inhibited the *H. pylori* growth with an MIC value ranging from 6.25 to 100 µg/mL, depending on the tested strain [24,25,26]. Paulo et al. showed that resveratrol could work as an *H. pylori* urease inhibitor, preventing the production of a local alkaline environment from the conversion of urea into ammonia, which allows the microorganism to survive to the stomach acidic conditions [26].

In general, resveratrol was found to be less active against Gram-negative bacteria (MIC values > 200 mg/mL) than against Gram-positive species. Efflux pump systems in Gram-negative species may be responsible for the decreasing susceptibility to resveratrol, as demonstrated by several experiments performed with mutants or in the presence of efflux pumps inhibitors. This observation suggested that the antibacterial activity of resveratrol could be partially due to the interaction with cytoplasmic or periplasmic targets in Gram-negative bacteria [11]. 

Furthermore, as Guo et al. [27] demonstrated, the antimicrobial activity of resveratrol may be related to the activation of the immune system response. Indeed, resveratrol and its dimethylated analogue pterostilbene (Figure 1) were found to activate the human cathelicidin antimicrobial peptide (*CAMP*) in both myeloid and keratinocyte cells. The human CAMP is expressed in immune and epithelial cells and it is able to kill a wide spectrum of bacteria. The human CAMP gene expression is mediated by 1α,25-dihydroxyvitamin D_3_ (1α,25(OH)_2_D_3_), litocholic acid, butyrate, and vitamin B3 [27,28]. When resveratrol and pterostilbene (10 µM) were combined with 1α,25(OH)_2_D_3_ (1 nM), they synergistically enhanced *CAMP* gene expression, representing a useful alternative to improve barrier defence and immune response against infections [27]. In another study [29] on the infection caused by the respiratory pathogen nontypeable *Haemophilus influenzae* (NTHi), leading to acute exacerbation of chronic obstructive pulmonary disease (AECOPD) [30], resveratrol resulted to be both an anti-inflammatory and antibacterial agent. Resveratrol displayed bacteriostatic effects on 14 genomically unrelated NTHi clinical strains at 175 µg/mL, without inducing antibiotic resistance in in vitro studies. Nonetheless, when resveratrol was assessed as pre-treatment in airway epithelial cells at sub-MIC, the NTHi cells invasions were significantly reduced. Regarding the immunomodulatory properties, Euba et al. demonstrated that resveratrol was able to lower IL-8 and hBD2 (bacteria-induced human β-defensin-2) gene expression in NTHi-infected A459 airway epithelial cells. In in vivo experiments on pulmonary NTHi-infected mice, the oral administration of resveratrol (150 mg/kg) reduced the bacterial load and the lung-inflammatory markers such as KC and TNF-α. To confirm these in vivo results in an alternative animal infection model, zebrafish was infected with NTHi and then treated by intraperitoneal administration of resveratrol (0.1 mg/g). The treated-zebrafish showed a significant decrease of *H. influenzae* c.f.u. (colony-forming units) and an increased survival with respect to those receiving perfusion solution-DMSO (1:1) [29]. Resveratrol was also studied as food preservative [31], evaluating the development of homologous (adaptation to the same products) and cross-resistance to different agents or stress conditions such as heat and acidic conditions, after exposure and adaptation of the foodborne pathogens *L. monocytogenes* and *S. aureus* to sub-MIC of resveratrol (MIC value 200 µg/mL and MBC value 400 µg/mL for both bacteria). Resveratrol adaptation of both *S. aureus* and *L. monocytogenes* did not result to induce homologous or cross-resistance to benzalkonium chloride and other tested antibiotics. However, an increase of MIC value of benzalkonium chloride from 2 to 4 µg/mL was observed in *L. monocytogenes* after eight sequential exposures to resveratrol (0.5 × MIC), which is likely due to the antioxidant properties of the polyphenol partially interfering with the oxidative stress induced by benzalkonium chloride. Studies were also performed to evaluate the tolerance to both heat and acidic conditions, which are treatments commonly applied in the food industry to eliminate microorganisms such as *S. aureus* and *L. monocytogenes*. An increment of survival rate of both studied microorganisms was observed upon adaptation to resveratrol and exposure to 55 °C and low pH (2.4), suggesting the role of resveratrol in the modification of cellular structures or protein synthesis leading to increased stress tolerance [31]. 

### 2.2. Natural and Synthetic Resveratrol Analogues 

Beyond resveratrol, in nature, there are several monomeric stilbenoids, differing in the position and number of hydroxy or methoxy groups at the two aromatic rings of the 1,2-diphenylethylene scaffold (Figure 1) [32]. The antistaphylococcal activity of a series of plant-derived resveratrol analogues was evaluated in in vitro studies against six ATCC (American Typical Culture Collection) strains and two clinical isolates of *S. aureus* (KI1 and KI2) [33] (Figure 1). Pterostilbene resulted to be the strongest growth inhibitor against all *S. aureus* strains, with an MIC value of 32 µg/mL, followed by piceatannol (MICs = 64–256 µg/mL), and pinostilbene (MIC = 128 µg/mL) (Figure 1, Table 1). Piceatannol was the most active compound. This finding confirmed previous evidence that the increasing number of hydroxy groups on phenolic compounds is associated with increasing toxicity to microorganisms [34]. However, since oxyresveratrol was found to be far less active than piceatannol, the position of the hydroxy groups was demonstrated to play another key role in the biological activity, according to previous observations [35]. Moreover, the number and the position of hydroxyl groups on ring B seemed to be more relevant for the inhibitory activity than the ones on ring A. In particular, *ortho*-dihydroxy groups on ring B significantly increased the antistaphylococcal effect. Conversely, on ring A, methoxy groups enhanced the antibacterial activity on *S. aureus* (3′-hydroxystilbene, pinostilbene, and pterostilbene), which decreased in the correspondent analogues with methoxy groups on the ring B (isorhapontigenin and rhapontigenin) [33]. This observation was further confirmed by a more recent study reporting the testing of a collection of resveratrol analogues and dimers against a panel of bacteria [36]. The permethylated and peracetylated monomers (i.e., trimethylresveratrol and triacetylresveratrol) were completely inactive, whereas pterostilbene displayed the strongest growth-inhibitor activity against *S. aureus* (MIC value = 4 µg/mL), along with its isomer 3,4′-dimethoxyresveratrol (MIC value = 64 µg/mL) (Figure 1, Table 2). Pterostilbene, bearing two methoxy groups on ring A and one hydroxyl group on ring B, resulted to be more active than the 3,4′-dimethoxy isomer. The same difference in the antibacterial activity between the two compounds was observed also against other Gram-positive bacteria, such as *L. monocytogenes* Scott A and *E. faecalis* DSM 20478, whereas 3,4′-dimethoxyreveratrol was more potent against *E. faecium* DSM 20477 and *B. cereus* DSM 9378. On the other hand, pinostilbene and desoxyrhapontigenin were the only monomers displaying a moderate activity against Gram-negative bacteria [36]. 

In 2019, Singh et al. [37] investigated the antibacterial activity of some natural stilbenoids and their synthetic analogues (Figure 1) against a panel of Gram-negative and Gram-positive bacteria. In the case of Gram-positive bacteria, 4′-bromo resveratrol, pinosylvin, pinostilbene hydrate, pterostilbene, and the dimer of 4,4′-dihydroxystilbene (Di-DHS) was shown to be more effective than resveratrol. In particular, pinosylvin and pterostilbene exhibited an MIC value of 25 µg/mL and Di-DHS exhibited an MIC value of 10 µg/mL against *S. aureus*. The antibacterial activity of 4′-bromo resveratrol and Di-DHS was directly correlated to oxidative stress, DNA cleavage, membrane damage, and physical perturbation, revealed by SEM (scanning electron microscope) analysis on *S. aureus*. In the case of Gram-negative bacteria, resveratrol resulted to be active against *Proteus vulgaris* MTCC 426 and *Salmonella typhimurium* MTCC 660 (inhibition: approximately 45% in both cases). All the other analogues were less potent than resveratrol, except for 4′-bromo resveratrol and pinosylvin, which showed higher inhibition than resveratrol against the two aforementioned Gram-negative bacteria and *Escherichia coli* BW25113 (inhibition >80%), whereas pinostilbene hydrate showed activity comparable to resveratrol. Experiments on Δ*tolC E. coli* (lacking efflux pumps) demonstrated that the poor activity on Gram-negative strains was due to the presence of efflux pump systems. Notably, all compounds were significantly more active against Δ*tolC* than against wild-type bacteria, except for 4,4′-dihydrostilbene (DHS), triacetylresveratrol, and trimethylresveratrol, which were inactive against all strains. Only 4′-bromo resveratrol and pinosylvin showed similar activity on Δ*tolC* and wild-type bacteria, presumably because they were poor substrates for the efflux pumps or able to act before being effluxed out. This study confirmed that hydroxy groups are fundamental for antibacterial activity. In fact, triacetylresveratrol and trimethoxyresveratrol were totally inactive. However, increasing the number of hydroxy groups did not increase the potency of piceatannol and oxyresveratrol, which conversely were less active than resveratrol. Furthermore, even if DHS and piceatannol did not show any relevant antibacterial activity, the most active compounds in this study (resveratrol, 4′-bromo resveratrol, pinostilbene, pterostilbene, Di-DHS) bear a substituent at the *para*-position, except for pinosylvin [37]. 

Pterostilbene was studied as an anti-biofilm agent against various opportunistic pathogens [38]. It displayed inhibitory activity on planktonic cells growth with an MIC_50_ (lowest concentration that did not allow visible growth of more than 50%) values of 18.60 and 25 µg/mL on *S. epidermidis* DMB 3179, *P. aeruginosa* NRRL B-59189, and *E. coli* DBM3125, respectively. In particular, pterostilbene showed a significant reduction of biofilm formation on *E. coli* (MAIC_50_, minimum adhesion inhibition concentration, = 40 µg/mL) and *S. epidermidis* (MAIC_50_ = 50 µg/mL), but it did not affect *P. aeruginosa* (MAIC_50_ > 170 µg/mL). However, pterostilbene was more effective in the pre-formed biofilm eradication of *S. epidermidis*, with an MBEC_50_ (minimum biofilm eradication concentration) of 25 µg/mL, than in the inhibition of biofilm formation [38]. 

Famuyiwa et al. [39] investigated the bioactivity of compounds extracted from the yellow inter-bulb of *Scilla nervosa* (Burch.) Jessop (*Hyacinthaceae* family), which is an important plant used in traditional medicine in Southern Africa to treat infections, inflammations, pains, constipation, and infertility [40]. Among the isolated compounds, only the stilbenoid isorhapontigenin (Figure 1) was found to display antibacterial activity, showing an MIC value of 19.53 µg/mL, but a high MBC value (312.50 µg/mL) against *Neisseria gonorrhoeae* (ATCC 49226) [39]. 

Rhapontigenin and desoxyrhapontigenin (Figure 1) extracted from the rhizomes of *Rheum tanguticum* Maxim. Ex Balf. (*Polygonaceae* family) displayed moderate in vitro antibacterial activity against various phytopathogens (Table 3) [41]. 

Pinosylvin (PS), pinosylvin monomethyl ether (PSMME), and dihydropinosylvin monomethyl ether (DHPSMME) (Figure 1), which were extracted from the knotwood and barks of different *Pinus* species, were tested against a panel of Gram-positive and Gram-negative bacteria (Table 4). DHPSMME showed the lowest activity, which was likely due to the absence of the double bond, which should mediate the electron transfer capability between the aromatic rings. Moreover, the two hydroxy groups at the *meta* position seemed to play a key role in the antimicrobial activity [42]. 

Studying secondary metabolites produced by *B. cereus* symbiotically associated with the entomopathogenic nematode (EPN) *Rhabditis* (*Oscheius*) sp. (a biocontrol agent against insect pests), Kumar et al. [43] isolated 3,5-dihydroxy-4-ethyl-*trans*-stilbene (ES) along with 3,5-dihydroxy-4-isopropyl-*trans*-stilbene (Figure 1). Confirming previous studies [44], ES was active only against Gram-positive bacteria, with MICs = 8–16 µg/mL on *S. aureus* MTCC 902 and *B. subtilis* MTCC 2756, whereas 3,5-dihydroxy-4-isopropyl-*trans*-stilbene inhibited also the growth of the Gram-negative *E. coli* MTCC 2622 (MIC = 8 µg/mL) [45]. In another study [46], 3,5-dihydroxy-4-isopropyl-*trans*-stilbene was isolated along with its epoxide (Figure 1), which was produced by *Photorhabdus luminescens*, an entomopathogenic gammaproteobacterium. Both compounds showed a good antibacterial activity against *B. subtilis* (NCIB3610) and *E. coli* (Nissle 1917) (MIC in the range 3–25 µg/mL) [46]. 

Piceatannol was found to display anti-biofilm activity against *S. mutans* at low micromolar concentrations (IC_50_ = 52 µM) through the inhibition of glucosyltransferases (Gtfs) [47]. *S. mutans* synthesises high-molecular weight glucosyl polymers by Gtfs to adhere to the tooth surfaces and to trap other oral bacteria and components contributing to the cariogenic environment development [48]. The compound did not inhibit the growth of commensal species such as *S. sanguinis* and *S. gordonii*, resulting in being highly selective for *S. mutans* biofilm. Moreover, piceatannol inhibited *S. mutans* colonisation in in vivo drosophila and rat models [47]. 

Sheng et al. studied the QS-inhibiting effects of 10 stilbenoids against *Chromobacterium violaceum* CV026 and *P. aeruginosa* PAO1, performing SAR studies [49]. Resveratrol, oxyresveratrol, and piceatannol displayed anti-QS activity against *C. violaceum* that was used as an indicator strain, since it produces violacein as a QS factor, creating a purple background. The presence of a white or cream halo around the well against the coloured background indicates QS inhibition. When the compounds lacking double bonds (phenantrenes or dihydrostilbenes) were tested, any QS inhibition was observed. Furthermore, pterostilbene was found to be inactive, highlighting the importance of hydroxy groups at the 3′- and 5′-position for the activity. Given that the active compounds were tested on *P. aeruginosa.* Resveratrol, oxyresveratrol, and piceatannol decreased the production of pyocyanin, which is a virulence factor controlled by QS at sub-MIC concentrations (400 µM). Moreover, the same three compounds significantly altered *P. aeruginosa* swarming motility at 100 µM dose [49]. 

Studying the extracts of *Ficus polita*, an edible plant from the family of Moraceae, largely used to treat infectious diseases, Kuete et al. identified (*E*)-3,5,4′-trihydroxy-stilbene-3,5-*O-β-D*-diglucopyranoside (Figure 1), which was tested against *P. smartii* ATCC29916, *P. aeruginosa* PA01, *K. pneumoniae* ATCC11296, *S. aureus* ATCC25922, *S. typhi* ATCC6539, *E. coli* ATCC8739, and *E.coli* AG100, displaying a moderate inhibitory activity (MIC in the range 64–256 µg/mL) [50]. 

**Figure 2:**
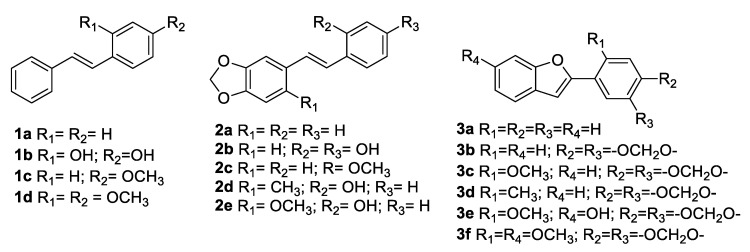
Structures of cicerfuran and synthetic analogues from Aslam et al. (2009) [52].

Cicerfuran (Figure 2, compound **3e**), a 2-arylbenzofuran belonging to the stilbene family, was first isolated from the roots of *Cicer bijugum*, which is a wild species of chickpea. Since it was reported to be produced as plant defence against *Fusarium oxysporum* f.sp. *ciceri*, Aslam et al. carried out the synthesis of cicerfuran along with other five 2-arylbenzofuran analogues and nine structural related stilbenes to investigate the antimicrobial activity and to perform SAR studies [51]. The synthesised compounds were tested against *B. subtilis* (IMI347329) and *P. syringae* (ATCC19310) (Table 5). Only compounds **1b**, **2b**, **2d**, **2e**, and cicerfuran **3e** (Figure 2) showed antibacterial activity with MIC values ranging from 25 to 100 µg/mL, highlighting the importance of the hydroxy function in the structure. Moreover, compounds **2d** and **2e**, bearing a methylenedioxy group as common structural feature, exerted similar activity, inhibiting the growth of both bacterial species. Conversely, compound **1b** was active only against *P. syringae* [52]. 

**Figure 3:**
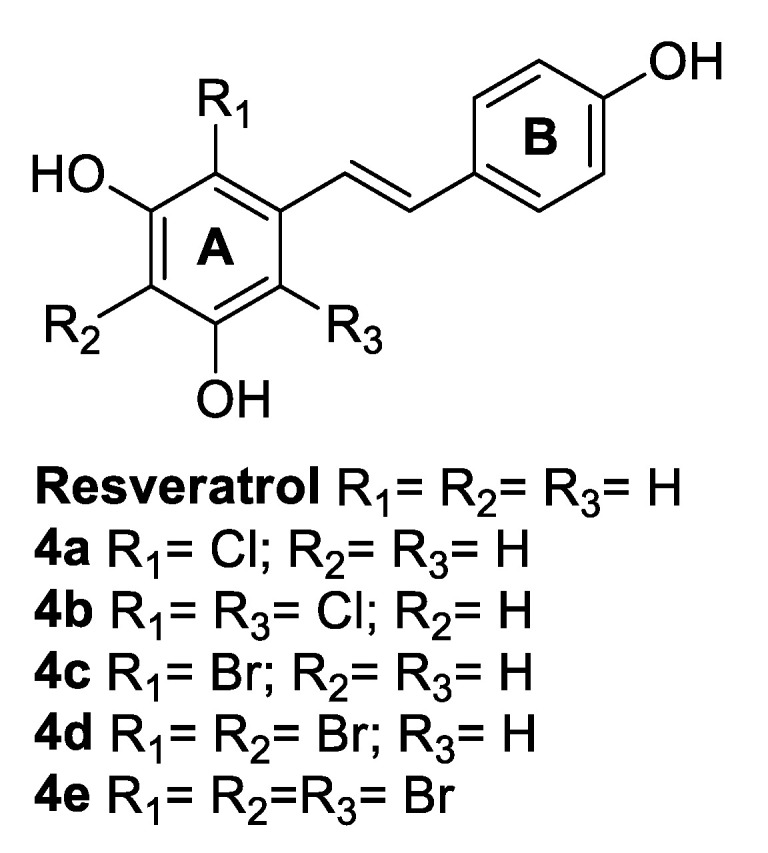
Structures of halogenated resveratrol derivatives from Li et al. (2012) [59].

Resveratrol was found to directly interact with myeloperoxidase (MPO) [53], which is a haem enzyme released by activated phagocytes during inflammation response. It catalyses the conversion of hydrogen peroxide into hypochlorous acid (HOCl) and hypobromous acid (HOBr), in the presence of physiological concentration of chloride and bromide anions, respectively [54]. The resulting hypohalous acids can act as antibacterial agents [55] and play a key role in the immune response, besides interacting with biological molecules, such as nucleic acids, proteins, and lipids, thanks to their antioxidant and electrophilic properties [56,57]. Furthermore, resveratrol was shown to significantly reduce the production of HOCl and nitric oxide (NO) produced by stimulated human neutrophils in a dose-dependent manner, acting on MPO [58]. Therefore, Li et al. [59] investigated resveratrol halogenated products, upon reaction with HOCl and HOBr, and their biological activities (Figure 3). The halogenated resveratrol derivatives were tested against *S. aureus* ATCC 25923 and *E. coli* ATCC 25922. Interestingly, the halogenation occurred only at the aromatic ring A, bearing two nucleophilic hydroxy groups, thus facilitating the electrophilic aromatic substitution. Resveratrol and its chloro-derivative **4d** were the most active against the Gram-positive *S. aureus*, but only compound **4d** maintained a good potency also against the Gram-negative *E. coli*, against which resveratrol resulted to be less active than all the halogenated derivatives synthesised (Table 6) [59]. 

To improve the antimicrobial effects of natural stilbenoids, several research groups focused on the synthesis of new derivatives containing the stilbene backbone. Many synthetic pathways to obtain modified stilbenoids have been investigated, such as aldol-type condensations, Perkin reactions, McMurry reactions, and recently the metal catalysed Mizoroki–Heck reactions [60,61].

**Figure 4:**
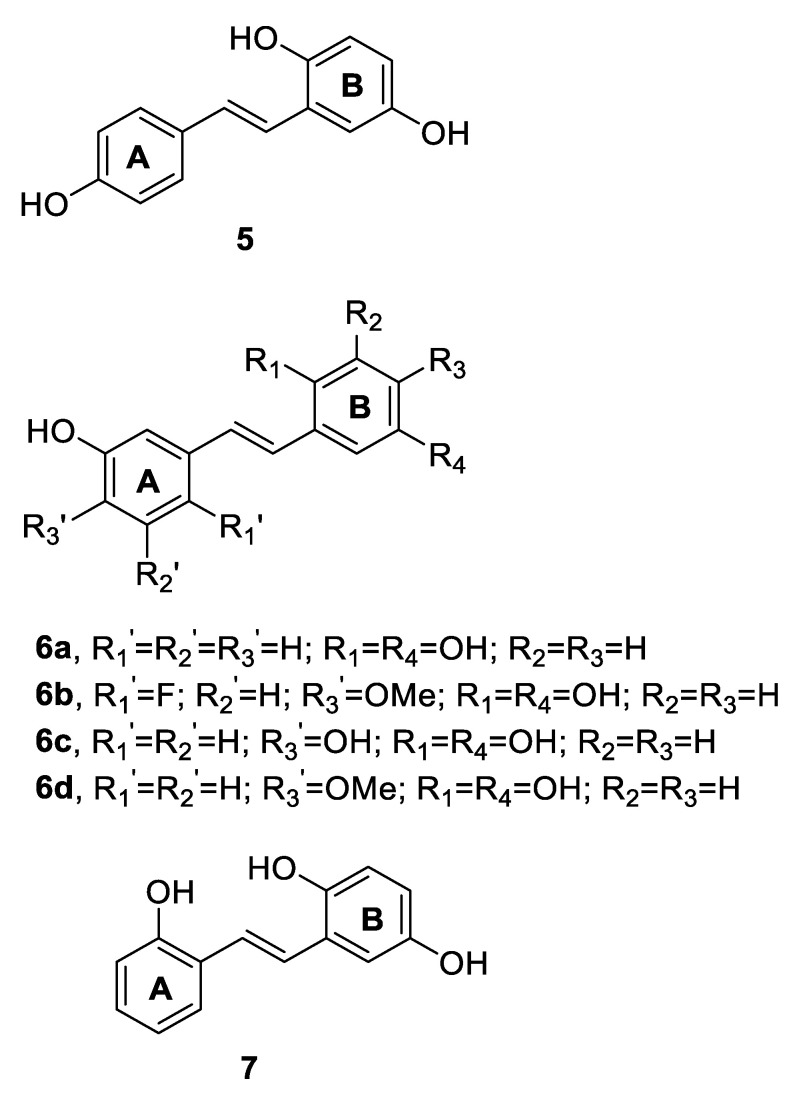
Synthetic stilbene derivatives from Albert et al. (2011) [62].

In 2011, by Mizoroki–Heck reaction, Albert et al. [62] prepared 25 compounds divided into three groups: 4-hydroxy stilbenes (e.g., compound **5**), 3-hydroxystilbenes (e.g., compound **6**) and 2-hydroxy stilbenes (e.g., compound **7**) (Figure 4). The compounds were tested in an agar diffusion assay for their antibacterial and antifungal activities. In particular, the stilbenoids reported in Figure 4 were the most active among the synthesised compounds, demonstrating significant growth inhibition of the Gram-positive *Bacillus subtilis* and *Bacillus brevis*, and the actinobacteria *Micrococcus luteus* (inhibition zone diameters ranging from 8 to 20 mm), even if less active than commercial antibiotics (streptomycin and tetracycline with inhibition zone diameters of 7–22 mm). All 25 molecules were inactive against the Gram-negative bacterium *Enterobacter dissolvens*. The data suggested that all stilbene derivatives needed two hydroxyl groups at the 2′ and 5′ positions on ring B to display antibacterial activity, if they were not mono-hydroxy substituted on ring A. Moreover, fluorine substituents enhanced the antibacterial effect, providing higher permeability of the compounds into the membrane [62]. 

### 2.3. Prenylated Stilbenoids 

**Figure 5:**
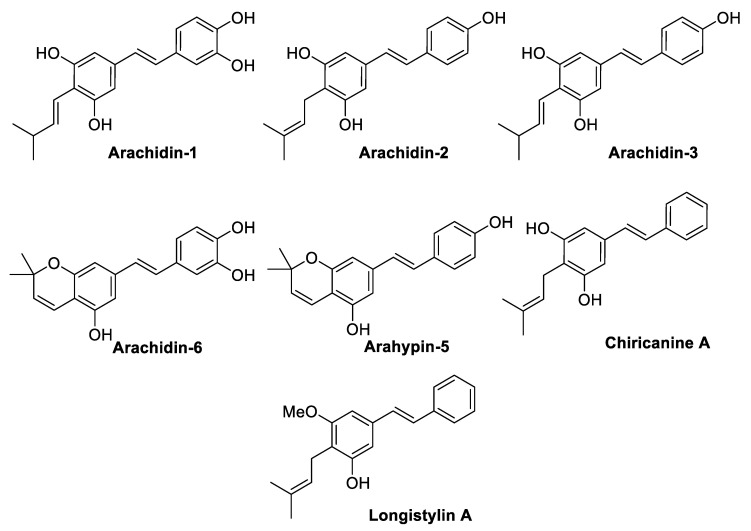
Structures of prenylated stilbenoids from de Bruijn et al. (2018) [68] and Wu et al. (2020) [69].

Prenylated stilbenoids are mainly isolated from the *Leguminosae* family, e.g., soybean, peanuts, mung bean, and their production can be stimulated by fungal elicitation. In particular, a prenyltransferase is responsible for attaching a prenyl-moiety to the phenol ring, and in the case of peanut stilbenoids, the prenylation usually occurs at the *para* position [63]. The prenyl-chain (3,3-dimethylallyl or 3-methyl-but-1-enyl substituent) may undergo further enzymatic cyclisation with an *ortho*-phenolic hydroxy group leading to five- or six-membered rings, which are furan and pyran derivatives respectively [64]. Since the prenylation of phenolic compounds increases in response to microbial attack in legume seeds, prenylated phenolic compounds are believed to have better antimicrobial properties than their non-prenylated precursors [65], and generally, this hypothesis has been confirmed [66,67]. This observation was traditionally explained as an effect of the enhanced hydrophobicity, which improves the affinity to biological membranes and the interaction with target proteins [64]. However, the specific position and the configuration of the prenyl chain seem to influence the bioactivity more than the hydrophobic effect [66]. De Bruijin et al. [68] isolated a series of prenylated stilbenes from an extract of *Rhizopus*-elicited peanut seedlings (*Arachis hypogaea*) and tested their antibacterial activity against *S. aureus* MRSA strain (18HN, spa type t034), using the corresponding non-prenylated precursors piceatannol, resveratrol, and pinosylvin as references to perform SAR analysis (Figure 5, Table 7). Chiricanine A was the most active molecule among the tested compounds. All the prenylated derivatives were more potent than their precursors, and within the tested compounds, the pyran ring enhanced the activity more than the prenyl chain. Nonetheless, the number of hydrogen bonds resulted negatively correlated with the anti-MRSA activity [68]. However, there was no correlation between the hydrophobicity and activity among the prenylated compounds, according to previous findings [67]. 

Wu et al. [69] evaluated the antibacterial activity of longistylin A (LLA) (Figure 5), an abundant prenylated stilbene isolated from the leaves of *Cajanus cajan* (L.) Millsp., which is commonly known as pigeon pea. The molecule was found to be inactive against *E. coli*, but it exerted significant antimicrobial activity against MRSA strains, displaying higher bactericidal activity (MBC) than vancomycin (Table 8). LLA antibacterial activity seemed related to a membrane potential dissipation and enhanced permeability, which induced cell lysis. Moreover, LLA was found to be effective as a topical agent to treat MRSA-infected wounds in mice by preventing further pathogens proliferation and reducing inflammation derived from the infection [69]. 

**Figure 6:**
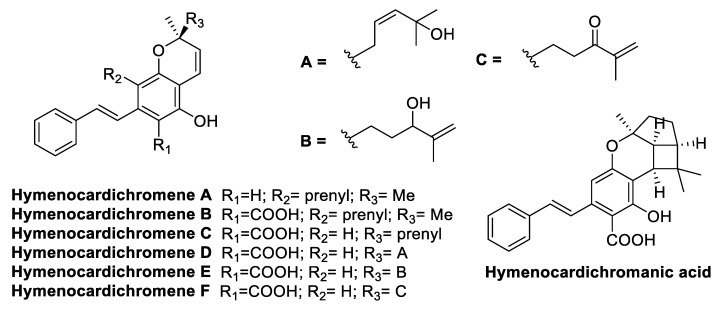
Structures of chromene and chromane stilbenoids Starks et al. (2014) [70].

A series of chromene and chromane stilbenoids was isolated from the leaves of *Hymenocardia acida* Tul. (*Phyllanthaceae* family), which is a small African tree used in traditional medicine to treat malaria, sickle-cell disease, cancer, and hypertension (Figure 6). The isolated stilbenoids were tested against MRSA-108 *S. aureus*, and they were found to display a moderate activity against the tested drug-resistant strain, showing hymenocardichromanic acid to be the most active compound (MIC = 8 µg/mL) (Table 9) [70]. 

### 2.4. Combretastatins and Their Analogues

**Figure 7:**
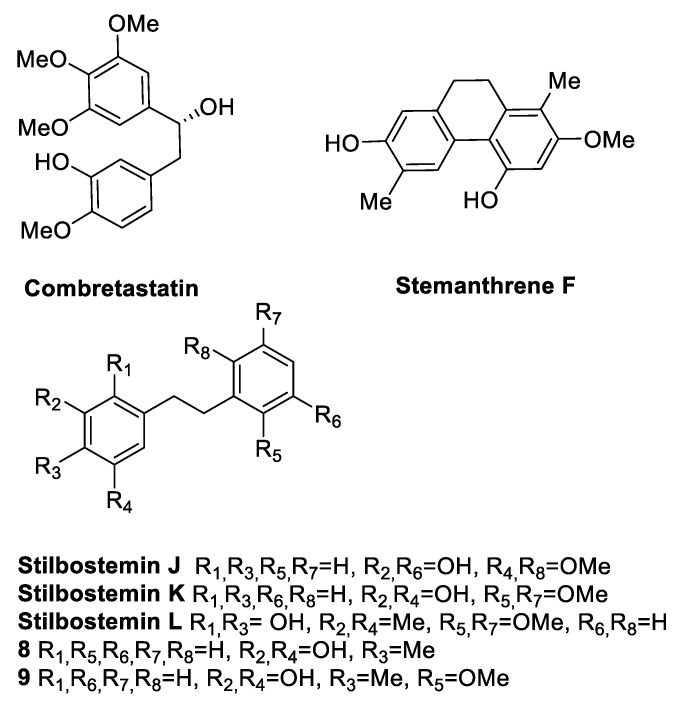
Structures of combretastatin from Shen et al. (2009) [32], stilbostemins and Stemanthrene F. from Yang et al. (2006) [71].

Combretastatins belong to the stilbenes family and include combretastatins, bibenzyls or dihydrostilbenes, phenanthrenes, and macrocyclic lactones. This subgroup of compounds was discovered in the African willow tree *Combretum caffrum* (Combretaceae), after the first isolation of combretastatin (Figure 7) [32]. Six stilbenoids extracted from the roots of *Stemona japonica* (Bl.) Miq, which is used in traditional Chinese medicine against insect pests and respiratory diseases, were tested against *S. aureus* (ATCC 25923), *S. epidermidis* (ATCC 12228)¸ and *E. coli* (ATCC 15628). Stilbostemin L, stemanthrene F, and compounds **8** and **9** (Figure 7) displayed significant antibacterial activity (MICs in the range 12.5–50 µg/mL) against the tested *Staphylococcus* strains (Table 10) [71]. 

**Figure 8:**
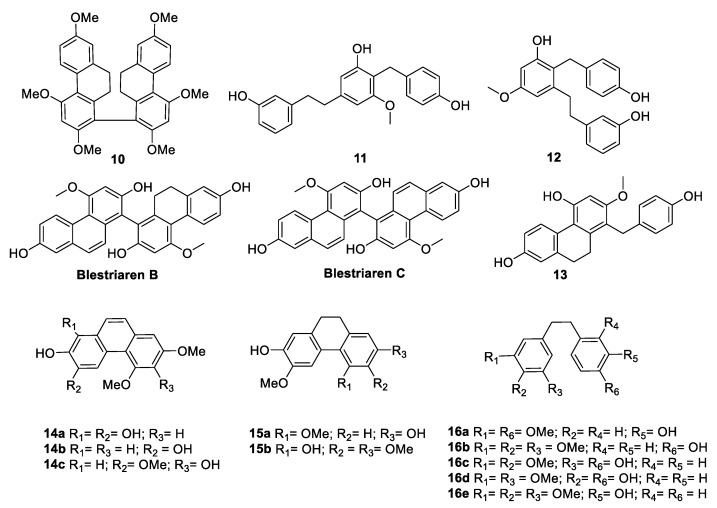
Structures of compounds isolated from *Bletilla yunnanensis* Schltr. from Yang et al. (2016) [72] and from *C. hereroense*, *C. collinum*, and *C. apiculatum* from Katerere et al. (2012) [73].

Yang et al. [72] isolated a new bisphenanthrene, 2,2′,4,4′,7,7′-hexamethoxy-9,9′,10,10′-tetrahydro-1,1′-biphenanthrene, along with other five known phenanthrene stilbenes (**10**–**13**, blestriaren B, blestriaren C) (Figure 8) from the tubers of *Bletilla yunnanensis* Schltr. (*Orchidaceae* family), which is largely present in China. The tubers of this plant have long been used to treat pulmonary diseases; thus, their chemical composition was investigated. The isolated compounds were tested against three Gram-positive bacteria, *S. aureus*, *S. epidermidis*, and *B. subtilis*, and two Gram-negative bacteria, *E. coli* and *Klebsiella pneumoniae*. Blestriarene B and C showed interesting MIC values (6.25–25 µg/mL) against *S. aureus* and *S. epidermidis* (Table 11) [72]. 

Katerere et al. [73] investigated the antimicrobial activity of a collection of stilbenoids (**14**–**16**, Figure 8) extracted from *C. hereroense*, *C. collinum*, and *C. apiculatum*, which are plants belonging to the African Combretaceae and used in Southern Africa to treat several disorders, mainly related to infections. The phenanthrenes **14a**, **14b**, **15a**, and **15b** showed moderate activity against *Mycobacterium fortuitum* and *S. aureus* (MIC = 25 µg/mL) (Table 12) [73]. 

**Figure 9:**
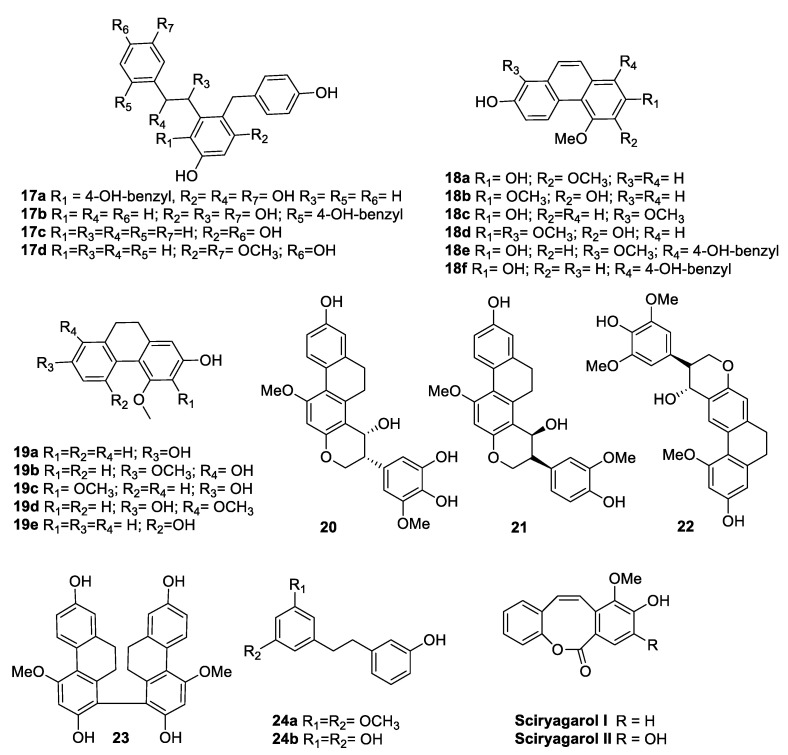
Structures of compounds extracted from *Bletilla striata* from Jiang et al. (2019) [74] and *Scirpus yagara* Ohwi from Liang et al. (2013) [76].

From the extract of *Bletilla striata* (Thunb.) Rchb.f. (Orchidaceae) [74], a plant used by traditional Chinese medicine to treat hematemesis, tuberculosis, malignant ulcers, traumatic bleeding, and cold [75], 21 compounds were isolated and evaluated for their antimicrobial activities against MRSA *S. aureus* ATCC 43300, *B. subtilis* ATCC 6051, *S. aureus* ATCC 6538, and *E. coli* ATCC 11775 (Figure 9, Table 13). Compounds **18a**, **19a**, **19c**, **20**, **21**, and **23** were shown to be active against *S. aureus* ATCC 6538 (MIC values ranging from 6 to 52 µg/mL). The methoxy groups seemed to decrease the antibacterial activity (compound **19a** vs **19c**), and the phenanthrenes were shown to be better than the corresponding dihydrophenanthrenes against MRSA *S. aureus* (compound **18a** versus **19a**) [74]. 

**Figure 10:**
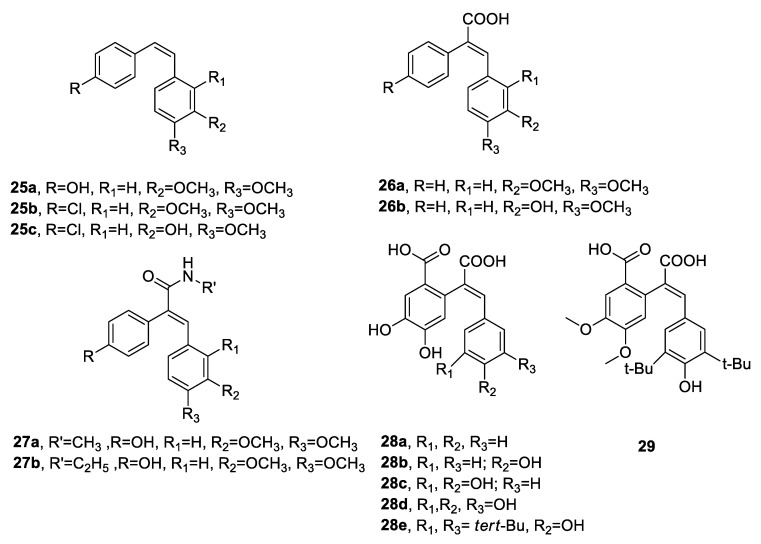
Structures of stilbene derivatives from Jain et al. (2015), Miliovsky et al. (2013), and More et al. (2015) [78,79,80].

Sciryagarol I and II (Figure 9) are two *cis*-stilbenoids isolated from the tubers of *Scirpus yagara* Ohwi (perennial Cyperaceae species), which is used in traditional Chinese medicine. The two compounds showed moderate antibacterial activity against *S. aureus* (MIC = 152 and 79.3 µg/mL, respectively,) whereas they were inactive against *E. coli* [76]. The natural stilbenoid combretastatin inspired the synthesis of numerous *Z*-stilbene derivatives, which are endowed with various biological properties [77]. Jain et al. [78] synthesised 30 novel *para*-(substituted phenyl)-2-(substituted phenyl) ethane compounds and evaluated their antibacterial activity against Gram-positive (*S. aureus*, *B. subtilis*) and Gram-negative (*P. vulgaris*, *E. coli*) strains. Some of the most active synthesised Z-stilbene compounds (**25a**–**c**, **26a**–**b**, **27a**–**b**) are reported in Figure 10. All compounds were moderately active compared to the standard ciprofloxacin, and 26b turned out to be the most potent molecule of the series against all four bacteria [78]. In 2013, a new one-pot diasteroselective synthesis of polyhydroxy Z-stilbenes was conceived by Miliovsky et al. [79] to prepare six new compounds. The antimicrobial activity of the novel molecules (**28a**–**e**, **29**, Figure 10) was evaluated. Compounds 28a–e did not show any antibacterial activity against the four bacterial strains tested, whereas the dimethylated derivative **29** exhibited 44% of the inhibitory effect of the standard amikacin on the growth of *B. subtilis* ATCC 6633 at the same concentration [79].

**Figure 11:**
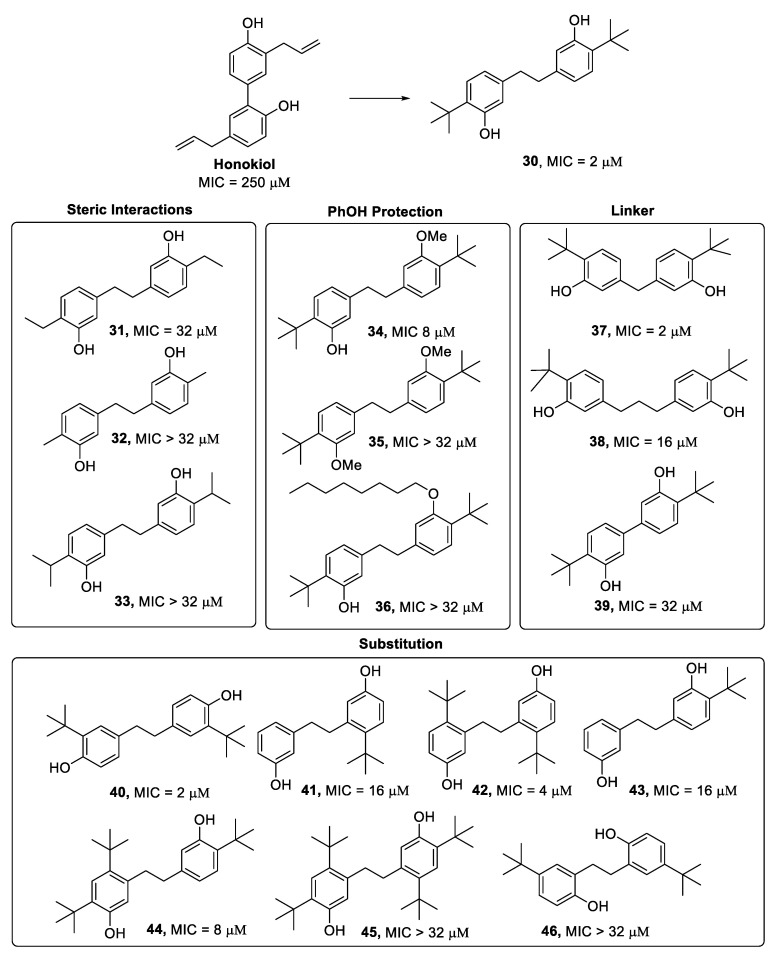
Structures of honokiol and analogues from Solinski et al. (2018) [82] and Ochoa et al. (2020) [83].

Inspired by the natural product honokiol, reported for its inhibitory activity against the oral bacterium *S. mutans*, and used in Chinese, Japanese, and Korean traditional medicine for centuries [81], Solinski et al. [82] carried out the synthesis of honokiol analogues (Figure 11). The dihydrostilbene **30** was identified to highly enhance the antibacterial effects of the natural precursor with an MIC value of 2 µM (66 ng/mL), and it revealed a strong bactericidal effect (MBC = 4 µM) on *S. mutans* [82]. In 2020, the same research group performed further SAR studies on **30**, designing 66 new analogs in order to understand the structural features crucial to the antibacterial activity on *S. mutans* planktonic growth. The MIC values of the synthesised compounds (Figure 11) revealed that alkyl groups on each aromatic ring were fundamental for the activity, and *tert*-butyl substituents in the number of two were found to be the optimum. Even in this case, the hydroxy functions resulted to be important structural features, since double alkylation of the phenolic groups caused a drastic drop in activity (**35**). However, the potency was retained when one hydroxy group was alkylated with a methyl group (**34**). Slight modification in the substitution pattern did not lead to any loss of activity (**40**, **42**), but the shift of the hydroxy groups to the *ortho* positions of the aromatic rings significantly decreased the potency (**46**), which was probably because of a misalignment of the hydrogen bonding postulated to play a key role in the interaction with the target. Regarding insight into the mechanism of action, **30** was found to interfere with the membrane stability and permeability without affecting membrane potential. Moreover, **30** showed a therapeutic index that was fourfold higher compared to cetylpyridinium chloride (CPC), which is a commercial antimicrobial used in toothpaste, mouthwashes, throat, and breath sprays, establishing the potential of further development of the dihydrostilbene **30** scaffold for oral care products [83]. 

**Figure 12:**
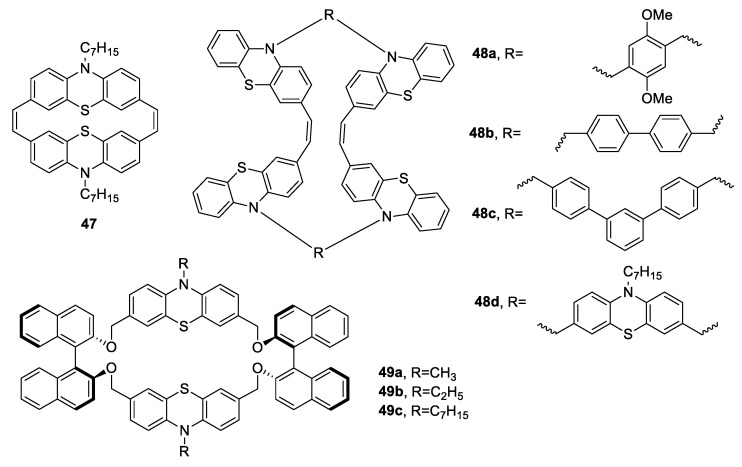
Structures of phenothiazinophanes from Kanagalatha et al. (2015) [87].

Recent studies have reported the preparation of cyclophanes constituted of phenothiazine units [84] with photoluminescence and electrochemical characteristics [85,86]. To obtain supramolecular systems with fluorescent sensing features useful in biology, Kanagalatha et al. [87] synthesised new phenothiazine-based fluorescent stilbenophanes (**47**, **48a**–**d**) and chiral phenothiazinophanes (**49a**–**c**) (Figure 12), and they tested them against a panel of bacteria (*S. aureus*, *S. pneumoniae*, *E. coli*, *K. pneumonia*, *P. vulgaris*, *S. typhi*, and *S. flexner*). The stilbenophane **48d**, with six phenothiazine units, was the best compound of the series, showing zone of inhibition diameters of 26.8 mm in *S. pneumoniae* (Gram-positive bacterium) and 23.4 mm in *K. pneumoniae* (Gram-negative bacterium) at the concentration of 50 µg/mL. This finding suggested a positive correlation between the increasing number of phenothiazine units and the antimicrobial effect [87]. 

### 2.5. Nitrogen and Sulfur-Containing Stilbene Derivatives

**Figure 13:**
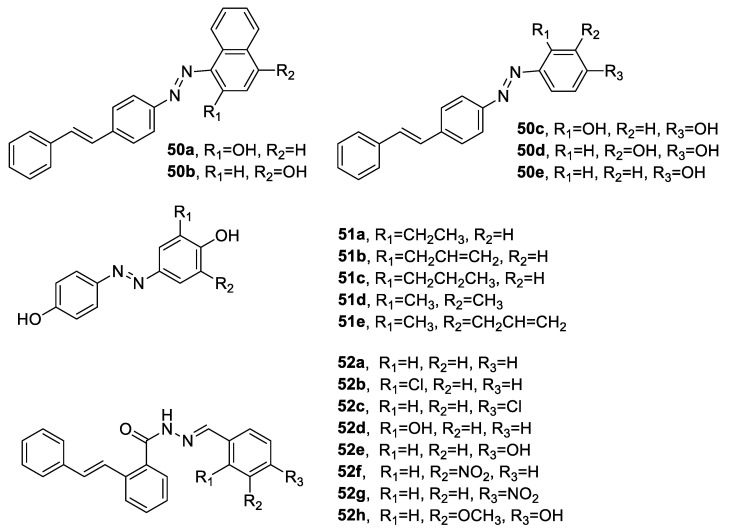
Nitrogen-containing stilbenes tested against Gram-negative and Gram-positive bacteria from Rezaei-Seresht et al. (2018) [88] and from Iqbal et al. (2018) [89].

Rezaei-Seresht et al. [88] evaluated the antibacterial activity of five novel azo dye-stilbene hybrid compounds, consisting of a phenolic moiety connected to a stilbene entity by an azo group (Figure 13), against eight Gram-negative bacteria (*Pseudomonas aeruginosa* ATCC27853, *Escherichia coli* ATCC25922, *Staphylococcus coagulase*, *Citrobacter frurdii* ATCC8090, *Enterobacter aeruginosa* ATCC21754, *Acinetobacer baumannii* ATCC13883, *Serratia marcescens* ATCC8100, *Klebsiella pneumoniae* ATCC13883) and one Gram-positive bacterium (*Streptococcus pneumoniae* ATCC 49619) by disc diffusion assay. Only the azo compounds **50c** and **50d** showed antibacterial efficacy against one Gram-negative *P. aeruginosa* with diameters of inhibition zones of 8.4 and 7.3 mm, respectively, and the Gram-positive *S. pneumoniae* (diameters of inhibition zones 9.7 and 10.1 mm, respectively). Interestingly, both active compounds bear two hydroxy groups, which presumably play a key role in the bacterial growth inhibition displayed by **50c** and **50d** [88].

**Figure 14:**
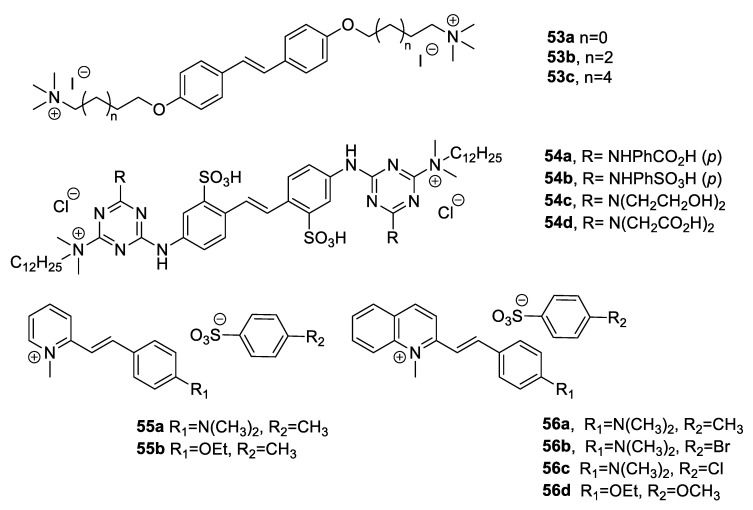
Chemical structures of ammonium salt-containing stilbenes from Zhou et al. (2018), Wan et al. (2017), and Chanawanno et al. (2010) [92,93,94].

Piotto et al. [90] designed and synthesised a small collection of azobenzenes, containing two nitrogen atoms in place of the olefinic carbons of the stilbenoids, in order to obtain novel compounds with higher antimicrobial activity and reduced toxicity. Five azobenzenes (**51a**–**e**, Figure 13), resulting from a virtual screening campaign and characterised by the lowest toxicity profiles in silico, were synthesised. The compounds were tested for the antimicrobial and antibiofilm activities to evaluate their application in polymer matrix for biomedical devices and food packaging. All the azo compounds showed higher antibacterial activity against *S. aureus*, *L. monocytogenes*, *S. typhimurium*, *P. aeruginosa* than resveratrol. Moreover, **51a**–**c** and **51e** were able to destroy more than 60% of pre-formed biofilms at a concentration of 30 µg/mL [90]. A small collection of eight *E*-stilbene azomethines (**52a**–**h**, Figure 13) was synthesised by Mizoroki–Heck reaction and tested for antimicrobial activity [89]. Compound **52g** was the most active compound of the collection, with MIC values of 0.22 mg/mL, 0.07 mg/mL, 0.10 mg/mL, and 0.06 mg/mL on *E. coli* (ATCC 25922), *S. aureus* (ATCC 25923), *K. pneumoniae* (ATCC 10031), and *B. subtilis* (ATCC 9637), respectively. Moreover, **52g** displayed stronger bactericidal effects than cefradine, the standard drug used as reference antibiotic. In addition, compound **52f**, bearing a nitro group at the *meta* position, exhibited good inhibition growth of the bacteria tested. These results highlighted the importance of electron-withdrawing groups, such as nitro group, for the antimicrobial effect in this series of compounds [89]. A novel class of molecules defined as conjugated oligoelectrolytes (COEs), which are constituted of a conjugate backbone and terminal polar pendant groups, was developed to mimic the strategic features of antimicrobial peptides (AMPs) [91]. Zhou et al. [92] designed and synthetised three CEOs (**53a**–**c**, Figure 14), consisting of a stilbene core linked to two terminal ammonium groups by alkyl chains of different length. The compounds were tested on *E. coli* K12 strain, the pathogenic *E. coli* UTI89, and on the Gram-positive *E. faecalis* OG1RF. The antimicrobial activity resulted to increase with increasing the alkyl chain length. The MIC values for *E. coli* K12 were 128, 16, and 4 µg/mL for **53a**, **53b**, and **53c**, respectively. Moreover, **53c** was active against both Gram-positive and Gram-negative bacteria and exhibited a high cell uptake of 72%, demonstrating a positive correlation between cell uptake and antimicrobial activity [92].

**Figure 15:**
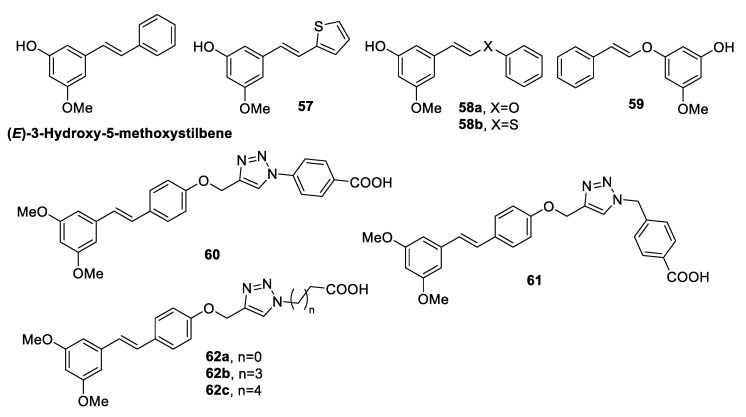
(E)-3-Hydroxy-5-methoxystilbene and synthetic analogues from Kabir et al. (2008) [96] and from Tang et al. (2019) [97].

Stilbene derivatives are optical whitening agents, absorbing invisible ultraviolet (UV) light at wavelengths below 400 nm and emitting violet-blue fluorescence in the UV-visible region, thus providing brightness and whiteness to a matrix [95]. However, stilbenes *cis*/*trans* isomerisation may occur upon exposure to sunlight, leading to fluorescence reduction. Therefore, Wan et al. [93] conceived novel stilbene derivatives bearing long-chain alkyl quaternary salts, in order to obtain optical whitening agents that are more stable to light and endowed with antibacterial activity. Four novel stilbene-12 alkyl quaternary ammonium salts, **54a**–**d** (Figure 14), were synthetised and tested for their optical whitening performance and for the antimicrobial potency against *S. mutans* UA159, *E. coli* ATCC 25922, and *C. albicans* 5313a. All the synthesised derivatives exhibited efficient whitening effect on cotton fiber, high fastness and stability to light, and good antibacterial activity. Compound **54d** resulted to be the most potent molecule, with MIC_50_ values of 16 and 4 µg/mL against *E. coli* and *C. albicans*, respectively [93]. In 2010, Chanawanno and colleagues [94] prepared other quaternary ammonium compounds (QACs) with a stilbene scaffold. A collection of 20 pyridinium and quinolinium stilbene benzenesulfonates was designed, synthesised, and tested for the antibacterial effects on the Gram-positive bacteria methicillin-resistant *S. aureus* (MRSA), *S. aureus*, *B subtilis*, vancomycin-resistant *E. faecalis*, *E. faecalis*, and on the Gram-negative bacteria *P. aeruginosa*, *S. typhi*, and *S. sonnei*. Besides the pyridinium and quinolinium rings, the synthesised derivatives differed in the ethoxy and dimethylamino substituents on the phenyl ring (Figure 14). All the compounds exerted antibacterial activity against at least one of the tested strains, indicating that they were more effective against Gram-positive than Gram-negative bacteria. The quinolinium and pyridinium moieties seemed to play a key role in the activity. Quinolinium headgroup-containing compounds were more potent than the pyridinium derivatives, especially against MRSA strains. In particular, **56a**–**c** were the most potent molecules with MIC values of 2.34 µg/mL against all tested Gram-positive microorganisms, indicating that they were more active than the standard quaternary ammonium disinfectant (benzalkonium chloride) and vancomycin. Conversely, **56a**–**c** were inactive against all Gram-negative bacteria, except for *S. sonnei*. Notably, quinolinium stilbene benzenesulfonates were 2–4 times more active than the corresponding iodide salts. In addition, the dimethylamino derivatives resulted to be more potent than the corresponding ethoxy-containing compounds [94]. The natural stilbenoid (*E*)-3-hydroxy-5-methoxystilbene (Figure 15), which has been isolated from the leaves of *Comptonia peregrina* (L.) Coulter, was found to exert antimicrobial activity against several clinically relevant Gram-positive bacteria, including MRSA and vancomycin-resistant enterococci (VRE) strains with MIC values of 32 and 16 µg/mL, respectively [96]. Therefore, Kabir et al. [96] performed SAR studies on (*E*)-3-hydroxy-5-methoxystilbene, synthesising, 22 analogues with modified aryl rings (**57**) or extended by a heteroatom to build (*E*)-phenoxystirenes (**58a**, **59**) and (*E*)-phenothiostyrenes (**58b**) (Figure 15). None of the analogs was effective against the Gram-negative *E. coli*, as the natural precursor. On the other hand, the natural stilbenoid and its analogs bearing a *meta*-hydroxy group (e.g., **57**, **58a**–**b**, **59**) exhibited good inhibitory effects against the Gram-positive bacteria MRSA, *S. aureus*, *B. cereus*, *M. smegmatis*, with MIC values ranging from 16 to 64 µg/mL, thus confirming the importance of at least one free hydroxy group for the antibacterial activity [96].

Considering the antibacterial activity of pterostilbene against methicillin-resistant *S. aureus* (MRSA) [98], Tang and colleagues [97] designed and synthesised a collection of 20 pterostilbene analogues linked to a -1,2,3 triazole moiety to increase the interactions with targets and to raise the water solubility of compounds [99]. The obtained derivatives (**60**–**62**, Figure 15), sharing a carboxylic acid moiety, were evaluated against MRSA and VISA (vancomycin-intermediate *S. aureus*) strains. All the compounds exhibited anti-MRSA activity, and 60 turned out to be the most active molecule, with MIC values in the range of 1.2–2.4 µg/mL and MBC values in the range of 19.5–39 µg/mL. SAR studies highlighted the importance of the presence of the carboxylic moiety linked by an appropriate spacer to the triazole ring. In particular, the activity improved by increasing the length of the alkyl chain as a spacer. The optimum of activity was reached when a phenyl group was used as a spacer. The active compounds were found to inhibit the activity of DNA polymerase in MRSA strains. Docking studies of compound **60** showed that the carboxylic group, the triazole moiety, and the oxygen of the phenol ring formed hydrogen bonds with Val320, Lys450, and Arg435 of DNA polymerase (PDB: 4b9t), whereas the pterostilbene scaffold was involved in hydrophobic interactions with Asp409, Pro424, Asp425, and Glu426 outside the DNA polymerase pocket. These findings rationalised the SAR and biological results obtained [97]. 

## 3. Oligostilbenoids

**Figure 16:**
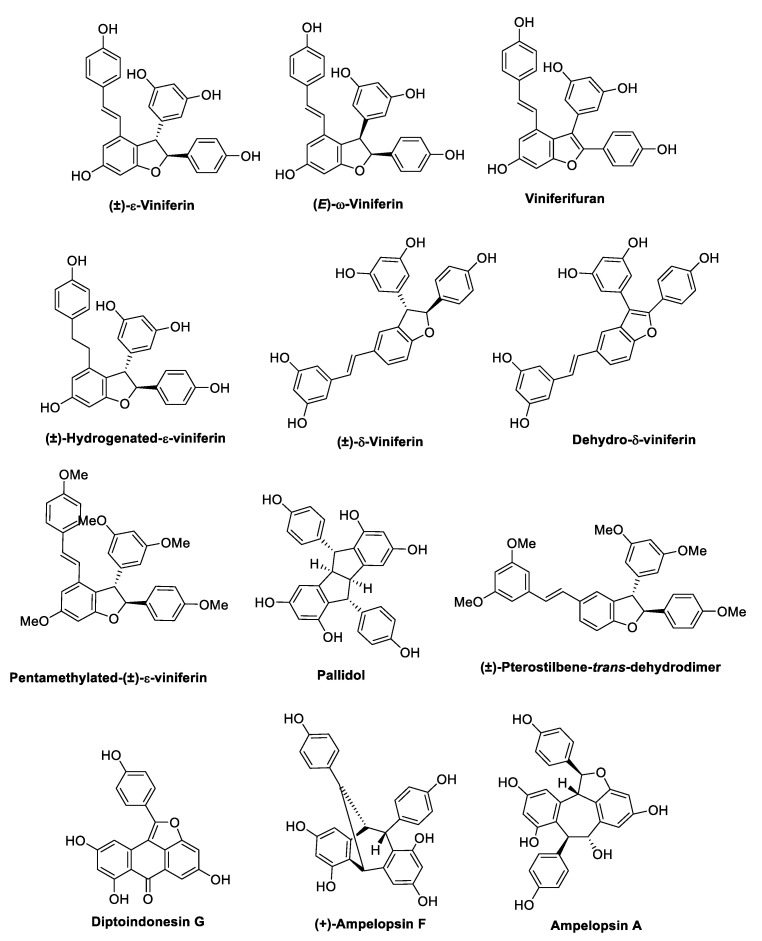
Structures of oligomeric stilbenoids from Shen et al. (2009) [32], Keylor et al. (2015) [100], and Sáez et al. (2018) [101].

**Figure 17:**
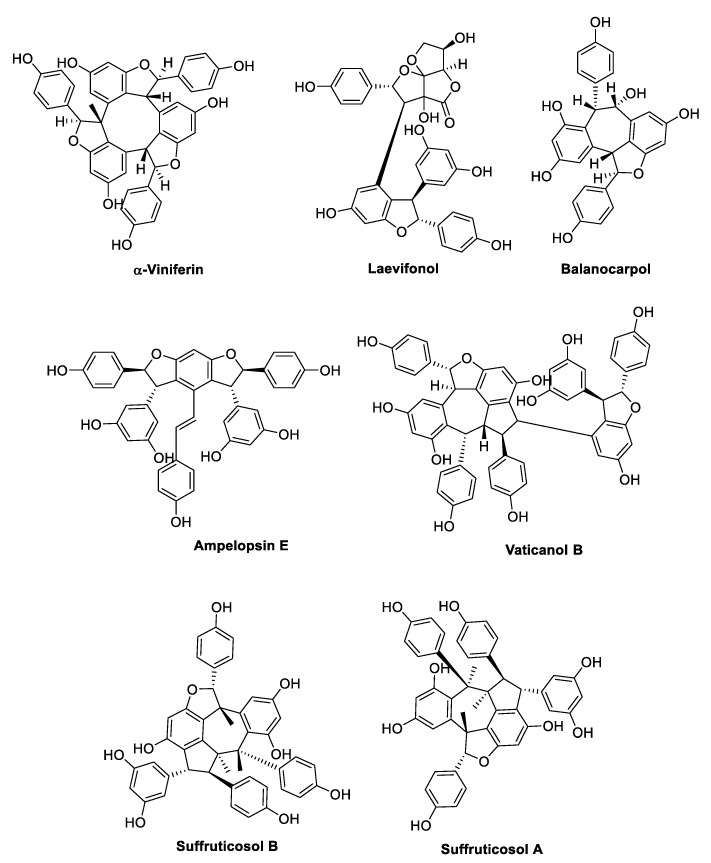
Structures of oligomeric stilbenoids from Shen et al. (2009) [32], Keylor et al. (2015) [100], and Sáez et al. (2018) [101].

**Figure 18:**
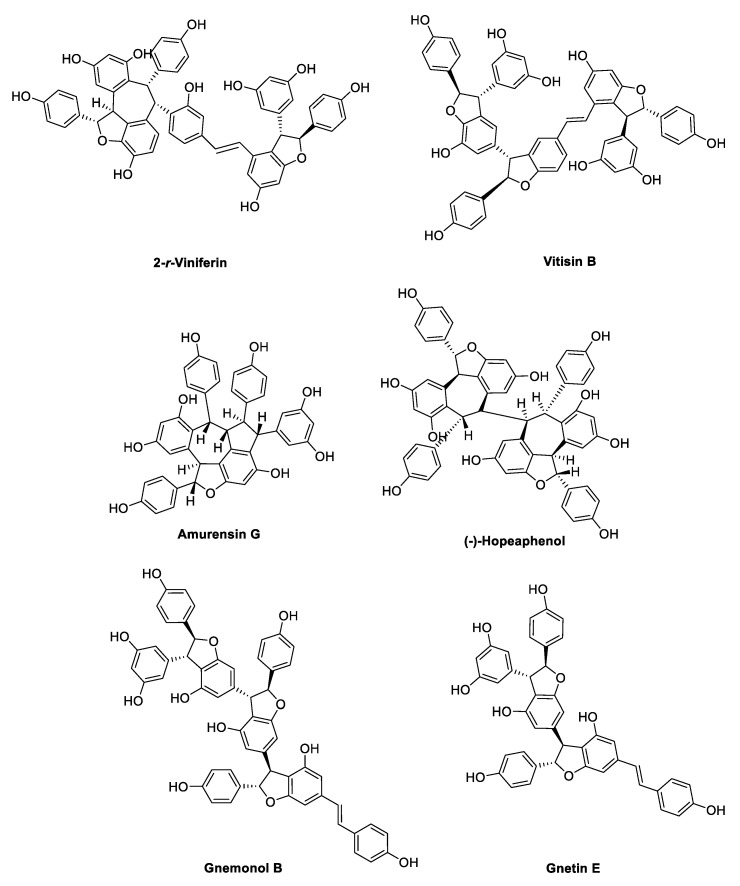
Structures of oligomeric stilbenoids from Shen et al. (2009) [32], Keylor et al. (2015) [100], and Sáez et al. (2018) [101].

**Figure 19:**
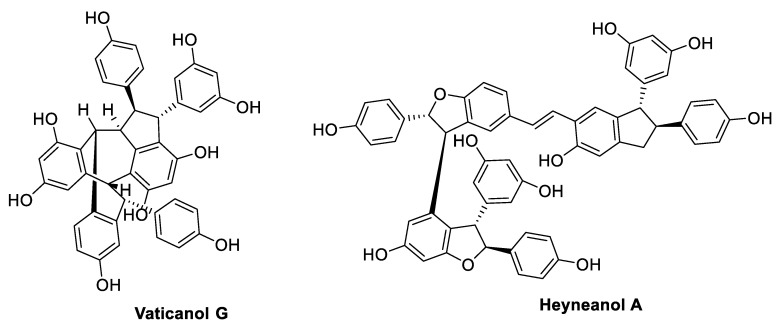
Structures of oligomeric stilbenoids from Shen et al. (2009) [32], Keylor et al. (2015) [100], and Sáez et al. (2018) [101].

Oxidative radical coupling and Friedel–Crafts reactions of two to eight units of resveratrol or other stilbene monomers yield more complex 3D structures, which occur in nature as dimers, trimers, tetramers, and higher-order oligomers (Figure 16, Figure 17, Figure 18 and Figure 19) [32,100,101]. 

Gnemonol B and gnetin E (Figure 18), isolated from gnetaceous plants, were tested against vancomycin-resistant enterococci (VRE) and methicillin-resistant *Staphylococcus aureus* (MRSA) strains, resulting in MIC values ranging from 6.25 to 25 µg/mL [102]. However, both compounds were found to be inactive against Gram-negative bacteria, including *E. coli*, *Proteus vulgaris*, *Serratia marcescens*, *K. pneumoniae*, and *P. aeruginosa* [103]. Peng et al. [104] discovered the potent antimicrobial activity of heyneanol A (Figure 19), which was isolated from the root extracts of the wild grape *Vitis thunbergii* (var. *taiwaniana*). In particular, heyneanol A was found to be remarkably active against MRSA strains with a MIC value of 2 µg/mL, which is comparable with that of vancomycin, besides exerting inhibitory growth effects on all tested Gram-positive bacteria (*Enterococcus faecium*, *S. aureus*, *S. agalactiae*, and *S. pyogenes*) with MIC values ranging from 2 to 4 µg/mL [104]. The resveratrol tetramer (−)-hopeaphenol (Figure 18), isolated from the leaf extracts of two Papua New Guinean rainforest plants, *Anisoptera thurifera* and *A. polyandra*, was investigated as an inhibitor of Type III secretion systems (T3SSs), constituting a conserved virulence system in Gram-negative pathogens [105]. Through T3SS action, bacteria are able to secrete and inject different virulence effector proteins into the host cell, inhibiting immunity response and facilitating the invasion and the proliferation of the bacteria into the eukaryotic cell [106]. In particular, at concentrations ranging from 3.3 to 100 µM, (−)-hopeaphenol inhibited dose-dependently and irreversibly the secretion of Yops (*Yerninia* outer proteins), the effector proteins of *Yersinia pseudotuberculosis*, likely covalently binding T3SS on the bacterial surface and without affecting the bacterial growth. At the concentration of 100 µM, the tetramer inhibited also T3SS of the superbug *P. aeruginosa* in infected HeLa cells and bound covalently to *Chlamydia trachomatis*, preventing the infection of pretreated HeLa cells. Notably, the compound did not affect the growth inhibition of any strain tested, confirming its activity as a selective inhibitor of the virulence T3SS [105]. 

Five resveratrol oligomers, including ε-viniferin (Figure 16), suffruticosol A, suffruticosol B (Figure 17), vitisin A, and vitisin B (Figure 18) were investigated for their antihemolytic activity on *S. aureus* [107]. The tetramer vitisin B was found to be the most active compound of the series, inhibiting hemolysis at 1 and 2 µg/mL by more than 70% and 95%, respectively. ε-Viniferin and vitisin A were found to be very effective at 10 µg/mL, whereas suffriticosol B and A were less potent [107]. From *D. lanceolate* stem barks, five oligomers were isolated: ε-viniferin (Figure 16), balanocarpol, α-viniferin, vaticanol B (Figure 17), and hopeaphenol (Figure 18) [108]. ε-Viniferin was found to be the most effective compound against *E. coli*, whereas balanocarpol was the most potent against *S. aureus* (Table 14). Both ε-viniferin and balanocarpol are stilbene dimers with a smaller molecular size than the trimer α-viniferin and the tetramers vaticanol B and hopeaphenol. Therefore, in this case, the molecular size seemed to cause a different penetration into the microorganism, affecting the antibacterial activity [108]. 

A series of oligostilbenes was extracted from the leaves of *Vitis amurensis* (Rupre.) Vitaceae [109] and evaluated for their antimicrobial activity against *S. mutans* and *S. sanguis* (Table 15). (+)-Ampelopsin A and F (Figure 16) did not show any antimicrobial effect, whereas ε-viniferin (Figure 16) was the most active compound with MIC values of 25 and 12.5 µg/mL against *S. mutans* and *S. sanguis*, respectively. Amurensin G and 2-*r*-viniferin (Figure 18) inhibited the *S. mutans* adherence at sub-MIC concentrations (total bacterial adherence inhibition, TBAI = 25 µg/mL, MIC = 50 µg/mL), which was consistent with the inhibitory activity on glucosyltransferases B and C (GTFs) exerted by the two compounds [109]. Indeed, GTFs synthesise the water-insoluble glucans, which mediate the sucrose-dependent adherence and accumulation of cariogenic streptococci [110]. Moreover, amurensin G showed a good inhibitory activity on the growth of both *S. mutans* and *S. sanguis* (MIC 50–12.5 µg/mL), whereas 2-*r*-viniferin was active only against *S. mutans*. Notably, ε-viniferin and amurensin G showed the same MIC as erythromycin [109]. 

Cho et al. [111] demonstrated the anti-biofilm properties of ε-viniferin (Figure 16) against the Gram-negative *P. aeruginosa* PA14 (a clinical isolate) and PAO1, and *E. coli* O157:H7 (ATCC43895), which is associated with a high risk of hemolytic–uremic syndrome when infections are treated with antibiotics. In particular, ε-viniferin at 50 µg/mL inhibited *P. aeruginosa* PA14 biofilm formation by 82%, and at 10 µg/mL inhibited *E. coli* O157: H7 biofilm formation by 98%, without affecting planktonic cells growth, thus reducing the risk of antibiotic resistance. However, further studies are required to elucidate the mechanism of action, since it did not appear to be related to virulence factors production, including pyocyanin, rhamnolipid, and pyochelin by *P. aeruginosa* [111]. In another study [112], (±)-ε-viniferin along with its stereoisomer (±)-*E*-ω-viniferin, the hydrogenated and the penta-methylated analogues (Figure 16), was tested on different *S. pneumonia* strains. (±)-ε-Viniferin was found to be the most active compound with an MIC value of 20 µM even on antibiotic-resistant strains. Therefore, the activity of (±)-ε-viniferin as an anti-biofilm agent was evaluated. The compound did not show any activity at sub-MICs, but at MIC concentration, it prevented biofilm formation in all strains tested, in contrast to aminoglycoside antibiotics, which cause stress in bacteria at sub-MICs, inducing biofilm formation [113]. Moreover, (±)-ε-viniferin inhibited bacteria and planktonic cells growth under biofilm, without a bactericidal effect. SEM analysis, crystal violet (CV) absorption assay, significantly increased release of total protein and genetic material (DNA and RNA), all supported alteration and lysis of bacteria cell membrane, thus suggested as the target of (±)-ε-viniferin [112]. δ-Viniferin and pallidol (Figure 16) were investigated for their antibacterial activity in several studies. δ-Viniferin showed interesting MIC values against the Gram-positive bacteria *B. cereus*, *L. monocytogenes*, and *S. aureus*, and against the Gram-negative *E. coli*, whereas pallidol was found to be inactive (Table 16) [114]. The presence of an efflux pump inhibitor significantly enhanced the activity against the Gram-negative bacterium, which was further confirmed by the increased susceptibility of the *E. coli tolC* (a key component of efflux systems in *E. coli*) deletion strain. Regarding the mechanism, δ-viniferin was demonstrated to downregulate two ABC transporters involved in cell division and the transport of molecules across the membrane. Moreover, from in vitro experiments and molecular modeling, δ-viniferin was found to be a strong inhibitor of DNA gyrase, replacing ATP from its binding site and thus preventing DNA replication [114]. 

In another study, δ-viniferin and other resveratrol dimers, including viniferifuran and dehydro-δ-viniferin (Figure 16), were found to display antimicrobial activity against a panel of Gram-positive bacteria, showing MIC values in the range of 1–16 µg/mL, whereas none of the compounds was active against Gram-negative bacteria at low concentrations (Table 17). In particular, dehydro-δ-viniferin, the most active compound, was demonstrated to display its activity against *L. monocytogenes* by membrane depolarisation and the loss of membrane integrity, leading to cytoplasmic membrane damage [36]. 

**Figure 20:**
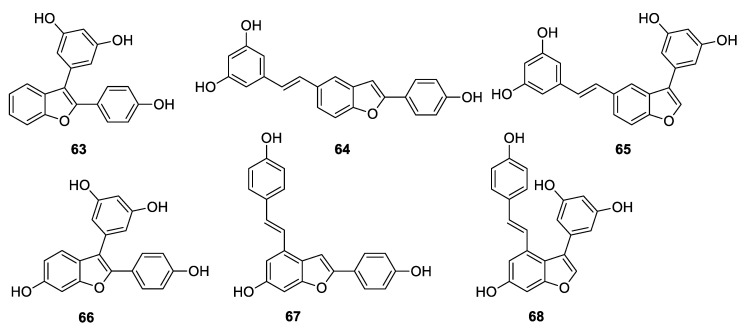
Structures of benzofuran derivatives of δ- and ε-viniferin from Catinella et al. (2020) [115].

In 2020, in order to perform SAR studies, Catinella et al. [115] synthesised a series of simplified derivatives of dehydro-δ-viniferin and viniferifuran by a systematic removal of the moieties linked to the benzofuran ring and evaluated the antibacterial activity against the foodborne pathogen *Listeria monocytogenes* Scott A (Figure 20). The simplified derivatives of dehydro-δ-viniferin (63–65) demonstrated a significant loss of the antibacterial efficacy compared to the parent compound, suggesting in this case the importance of all three phenolic rings linked to the benzofuran core for the activity. Conversely, the structural simplification of viniferifuran (MIC = 16 µg/mL, MBC > 512 µg/mL) led to compound 67 with improved activity (MIC = 8 µg/mL; MBC = 64 µg/mL) against *L. monocytogenes*, which is comparable to the standard chlorhexidine. These data confirmed that the shape, the geometry, and the relative position of the hydroxy groups on the aromatic rings significantly affect the antimicrobial potency [115].

## 4. Combination Therapy

A combination of antibiotics is an alternative strategy to fight antibiotic resistance, widening the antibacterial spectrum compared to the monotherapy. Bioactive adjuvants with weak or no antibacterial activity, such as efflux pump inhibitors, membrane permeabilisers, and bacterial enzyme inhibitors may increase the potency of primary antibiotics, such as the β-lactamase inhibitor clavulanic acid administered with amoxicillin. On the other hand, the combination of two or more antibiotics may act on multiple targets of pathogens, which are assumed to have more difficulty to resist to diverse attacks [17,116]. Nonetheless, additive or synergistic effects of the combination therapy may avoid undesirable side effects of individual antibiotics due to high dose regimens [117]. 

In 2012, resveratrol and 3,5-dihydroxy-4-isopropylstilbene (Figure 1) were tested in combination with ciprofloxacin and cefotaxime against the Gram-positive bacteria *B. subtilis* MTCC 2756 and *S. aureus* MTCC 902, and the Gram-negative bacteria *E. coli* MTCC 2622 and *P. aeruginosa* MTCC 2642 by checkerboard microdilution test (MDT) and time-kill-assay (TKA) [118]. Both compounds resulted to be synergistic with ciprofloxacin against either Gram-negative or Gram-positive bacteria, whereas they demonstrated additive effects with cefotaxime. Therefore, the combination with a fluoroquinolone broadened the antibacterial spectrum of stilbenoids, which are usually found to be inactive against Gram-negative and anaerobic bacteria, besides lowering the dose of ciprofloxacin [118].

Another study [119] showed promising results of the combination of resveratrol with benzoyl peroxide in in vitro experiments on *Propionibacterium acnes*, which is the causative agent of acne. Benzoyl peroxide is highly effective as an anti-acne agent, but its side effects limit its use. On the other hand, resveratrol inhibits the *P. acnes* growth with an MIC value of at least 50 µg/mL, with low bactericidal activity. The combination treatment revealed a high initial antibacterial activity due to benzoyl peroxide, followed by the longer lasting inhibitory effects shown by resveratrol alone. By transmission electron microscopy, structural alteration of bacterial membranes was noted with intramembranous edema and the loss of extracellular fimbrial structures in the presence of resveratrol. Therefore, thanks to the *P. acnes*-enhanced susceptibility due to resveratrol bacterial membrane alteration, the combination treatment may reduce the side effects of benzoyl peroxide, allowing a lower concentration with respect to that used in the current treatment. However, it should be stressed that in vivo studies are still needed [119].

In a more recent study [120], resveratrol was found to display a synergistic activity with colistin, which belongs to polymixins, last-resort agents against severe infections by multidrug-resistant Gram-negative bacteria. Resveratrol alone was ineffective against all tested strains. However, checkerboard assays demonstrated a dose-dependent synergism between resveratrol (used at a concentration ranging from 8 to 128 µg/mL) and colistin on colistin-resistant (COL-R) strains, including intrinsically resistant species such as *S. marcescens* and *P. mirabilis* (Table 18). Moreover, the time-kill assays revealed that the combination of resveratrol (128 µg/mL) with colistin (2 µg/mL) was bactericidal for 11 out of the 15 COL-R strains tested, whereas the bactericidal effect on the remaining strains required colistin at 0.5 × MIC or 1 × MIC, except for *E. coli* FI-4531 that did not seem to be susceptible to the combination. The synergism mechanism remains unknown, but the susceptibility of COL-R strains and the lack of any synergistic activity of colistin-susceptible (COL-S) strains suggested that resveratrol may interact with the lipid A modification systems that are involved in the resistance of COL-R strains [120]. 

Stilbenoid dimers and trimers were evaluated for their potential interaction with vancomycin and linezolid against MRSA strains (ATCC 33591 and HUKM strains) [121]. ε-Viniferin (Figure 16) reduced the MIC values of vancomycin by eightfold and 16-fold against ATCC and HUKM strains, respectively, demonstrating a synergistic effect. However, no synergistic interaction was observed between ε-viniferin and linezolid. Moreover, ε-viniferin resulted to antagonise the bactericidal activity of vancomycin, in spite of their synergistic bacteriostatic activity [121]. This finding may confirm ε-viniferin as a bacteriostatic anti-MRSA agent [117], since it has been already reported that a bactericidal agent is usually more efficient on multiplying bacteria [4]. Gnemonol B (Figure 18) exhibited a partial synergism with gentamycin against MRSA strains, and with ampicillin, gentamycin, micocycline, fosfomycin, or vancomycin against VRE strains. Gnetin E (Figure 18) was partially synergistic with gentamycin, minocycline, fosfomycin, and vancomycin against MRSA, and with gentamycin and vancomycin against VRE [103].

## 5. Delivery Systems

As showed in the previous paragraphs, stilbenoids exert antibacterial activity on several microorganisms with a variety of mechanisms of action. However, their industrial application as food preservatives or their use in clinical treatments have been limited by their poor availability and bioaccessibility, due to low water solubility, high metabolism, chemical instability, and interactions with food matrix such as proteins and fats [122,123,124]. Therefore, in the last years, efforts have been made to find delivery systems for stilbenoids, especially for resveratrol, for application as antibacterial agents in humans as well as preservatives in food and pharmaceutical packaging. A useful approach is the development of nanoparticles (NPs) as delivery systems [123,125]. Glaser et al. [126] combined the antimicrobial activity of chitosan, a polysaccharide biopolymer derived from the partial N-deacetylation of the natural chitin [127], and the antioxidant and antibacterial potency of resveratrol, to develop a coating for polyethylene (PE) and polypropylene (PP) polymer foils for pharmaceutical and food packaging [126]. The first layer consisting of 2% chitosan dispersion conferred antimicrobial properties, which are mainly due to the positively amino groups of chitosan [127], whereas the uppermost layer, containing nanoparticles of chitosan and resveratrol, enabled antioxidant and antibacterial activities. In particular, the obtained results showed that an antimicrobial efficacy (calculated as the percentage of reduction, which is the difference between the viable cells recovered after the incubation of untreated material and the viable cells recovered after the incubation of treated material) of more than 90% and 75% was achieved against *S. aureus* and *E. coli*, respectively [126]. In order to improve the low bioavailability of phenolic compounds, Vitonyte et al. [128] combined resveratrol and gallic acid in liposomes modified by the addition of a co-solvent, propylene glycol or glycerol, to produce Penetration Enhancer-containing Vesicles (PG-PEVs) and in glycerosomes as effective pharmaceutical tools against skin pathologies associated with oxidative stress and bacterial infections. The vesicles system formulation prevented the potential cytotoxic effect of high concentrations of the two active ingredients and improved cell viability after hydrogen peroxide-induced stress, in comparison to the effect of the dispersion of the phenols in water/glycerol blend (1/1). Overall, propylene glycol liposomes (PG-PEVs) and glycerosomes increased the total phenol accumulation in dermis. Indeed, glycerol promoted resveratrol penetration of the stratum corneum bilayer. On the other hand, phospholipid vesicles significantly enhanced the deposition of the hydrophilic gallic acid in the whole skin, as compared to the deposition obtained by the water/glycerol dispersion. Regarding the antimicrobial effects, all vesicle formulations showed good antibiofilm activity against *Streptococcus intermedius* and *Streptococcus pyogenes* (MBIC, minimum biofilm inhibition concentration = 312 µg/mL) [128]. 

In 2015, Silva et al. [129] assessed the antimicrobial activity of pinosylvin and pterostilbene (Figure 1), encapsulated in cyclodextrin inclusion complexes (ICs), against *Campylobacter jejuni* and *Campylobacter coli*, causing bacterial foodborne diarrhoeal diseases. The study showed that pterostilbene as pure compound exerted no antibacterial activity against the reference strains (ATCC 33560 and ATCC 33559) and moderate activity on the clinical isolates *C. jejuni* 930/12 and *C. coli* 22/08 (MIC value = 50 µg/mL). However, the pterostilbene ICs resulted in MIC values fivefold higher than those of the pure compound. Conversely, the antibacterial activity of pinosylvin as pure compound (MIC values ranging from 25 to 50 µg/mL) was very similar to that exerted as IC (MIC values ranging from 16 to 64 µg/mL). In particular, the value of the apparent stability constant (K_S_) of pterostilbene ICs was several orders of magnitude higher than that of pinosylvin ICs, indicating a more difficult dissociation of pterostilbene from the cyclodextrin complex, which is necessary for the antimicrobial compound to act inside the bacterial cell [130]. Investigating the pinosylvin ICs mechanism of action, the bacterial membrane was postulated to be the main target by four steps, including diffusion and collision of the IC with the membrane surface, followed by the complex dissociation and release of pinosylvin from the IC cavity and final diffusion through the bacterial membrane [131]. Furthermore, the pinosylvin ICs inhibited *Campylobacter* growth even at 4 and 20 °C, causing membrane depolarisation, increased permeability, and the inhibition of an efflux pumps system [129]. The effect of metallic nanoparticles on the antimicrobial activity of resveratrol and vice versa was also studied (Table 19) [132]. Gold nanoparticles (AuNPs) and silver nanoparticles (AgNPs) have demonstrated antibacterial properties through multifaceted mechanisms that are believed to avoid the bacterial resistance [133]. Therefore, novel nanoparticles carrying resveratrol on gold (Res-AuNPs) and on silver ions (Res-AgNPs-NaOH and Res-AgNPs-SDS, combined with sodium dodecyl sulfate) were developed and tested against 22 strains of Gram-positive and Gram-negative bacteria. The results showed that overall, resveratrol associated to all kind of metallic nanoparticles was more effective than resveratrol alone. The Res-AgNPs-SDS were twofold more potent than Res-AgNPs-NaOH against *S. aureus* (SG511, 285 and 503), *E. coli* DC 2, *Klebsiella oxytoca* 1082E, and *S. pneumoniae. Streptococcus pneumoniae* was the most sensitive microorganism, and the Res-AuPN nanocarriers further lowered the MIC value from 28.5 to 14.25 µg/mL [132]. Even if the antibacterial mechanism of these metallic nanocarriers still needs to be elucidated, several hypotheses have been proposed: (1) silver NPs bind to sulfhydryl and disulfide groups of bacterial membrane constituents, hampering cell wall synthesis; (2) positive charged metals electrostatically interact with the negative charged phospholipid bilayer of bacterial membranes, disrupting cell membrane potential and integrity; (3) metal NPs bind to DNA or to cytosolic proteins, such as enzymes involved in the respiratory chain and other metabolic pathways; and (4) metals from NPs can produce ROS, damaging DNA, proteins, and cell walls [133]. 

Resveratrol-biopolymers, deposited as thin, uniform, and adherent coatings through matrix-assisted pulsed laser evaporation (MAPLE), were prepared in order to incorporate the natural compound in bulk materials to avoid bacterial adhesion and subsequent infection [134]. The biopolymers were investigated for their antimicrobial efficiency against both Gram-positive and Gram-negative strains. The film consisting of resveratrol combined with PVP (polyvinylpyrrolidone) resulted to be effective against *S. aureus* strains, mainly by a contact-killing effect, which hampered initial bacterial adherence through physicochemical interactions or bactericidal effects on the planktonic cells. Biofilms containing only PVP showed activity similar to that of PVP–resveratrol combination, which was presumably because of the anti-fouling effect of PVP that guaranteed a hydrophilic environment, impeding the hydrophobic interactions necessary for the initial bacterial adhesion [134]. 

## 6. Conclusions

Stilbenoids represent a class of natural products endowed with several biological activities. Having evolved over several millennia to acquire specific ligand–protein binding motifs, these privileged scaffolds are biologically prevalidated platforms in the search for new active compounds. The most studied stilbenoid is resveratrol, which has received massive attention for its potential health benefits, including the management and prevention of infectious diseases. However, in recent years, also resveratrol-derived monomers, dimers, and oligomers have been extensively studied for their antimicrobial activity. The interest in the pharmacological potential of resveratrol-derived compounds has increased due to both poor bioavailability and incomplete understanding of the pharmacodynamics of resveratrol, which severely hamper its therapeutic applications. In this review, we have provided a summary of the most recent studies concerning the antimicrobial activity not only of the parent compound, but also of other natural stilbenoids, which in most cases were found to be more active than resveratrol itself. Synthetic approaches and SAR studies to give agents with better pharmacokinetic profiles and an improved spectrum of antimicrobial activity were reported as well. Despite the difficulties in comparing the obtained results, it is clear that resveratrol-derived stilbenoids possess promising activity, which has been mainly confirmed by in vitro studies. Nevertheless, a great effort to carry out in vivo experiments is still needed and remains of primary importance to confirm the antimicrobial potential of stilbenoids for future clinical applications. 

## Figures and Tables

**Table 1:** In vitro growth-inhibitory effect of stilbenoids against *S. aureus* from Zakova et al. (2018) [33].

Compound	Strain Tested/MIC (µg/mL)							
	ATCC 43300	ATCC 25923	ATCC BAA 976	ATCC 29213	ATCC 33591	ATCC 33592	KI1	KI2
3′-Hydroxypterostilbene	256	128	256	128	128	128	256	256
Isorhapontigenin	128	256	256	256	256	256	256	256
Oxyresveratrol	256	256	256	256	256	256	256	256
Piceatannol	64	64	64	64	64	64	64	256
Pinostilbene	128	128	128	128	128	128	128	128
Pterostilbene	32	32	32	32	64	32	32	64
Resveratrol	>512	256	>512	>512	>512	256	>512	>512
Rhapontigenin	256	256	256	256	256	128	256	256
Oxacillin *	16	0.125	8	0.125	128	64	1	16

MIC: minimum inhibitory concentration; ATCC: American type culture collection; KI: clinical isolates. * Represents reference control.

**Table 2:** MIC and minimum bactericidal concentration (MBC) values (in brackets) of stilbenoid monomers from Mattio et al. (2019) [36].

Compound	MIC (MBC) µg/mL					
	*S. aureus* ATCC 25923	*P. aeruginosa* ATCC 27853	*L. monocytogenes Scott A*	*E. faecium* DSM 20477	*E. faecalis* DSM 20478	*B. cereus* DSM 9378
Resveratrol	512 (>512)	> 512 (>512)	-	-	-	-
Pterostilbene	4 (128)	512 (512)	64 (128)	32 (512)	32 (128)	16 (512)
Piceatannol	>512 (>512)	128 (>512)	-	-	-	-
3′-Hydroxy-pter.	128 (512)	128 (>512)	-	-	-	-
Trimethoxy-res.	>512 (>512)	> 512 (>512)	-	-	-	-
Triacetoxy-res.	>512 (>512)	>512 (>512)	-	-	-	-
3,4′-Dimethoxy-res.	64 (512)	128 (256)	256 (512)	4 (512)	128 (512)	8 (>512)
Desoxy-rhapontigenin	256 (>512)	64 (>512)	-	-	-	-
Pinostilbene	512 (>512)	64 (>512)	-	-	-	-

**Table 3:** MIC of stilbenoids against phytopathogenic microorganisms from Pham et al. (2017) [41].

Compound	MIC (µg/mL)					
	*Acidovorax avenae subsp. cattlyae*	*Agrobacterium tumefaciens*	*Burkholderia glumae*	*Clavibacter michiganensis subsp. michiganensis*	*Pectobacterium carotovora subsp. carotovora*	*Pseudomonas syringae pv. actinidiae KW11*
Rhapontigenin	150	150	75	150	300	–
Desoxyrhapontigenin	38	75	38	75	–	38

**Table 4:** Antibacterial activity of pinosylvins from Välimaa et al. (2007) [42]. DHPSMME: dihydropinosylvin monomethyl ether, PS: pinosylvin, PSMME: pinosylvin monomethyl ether.

Compound	Inhibitory Effect (Percentage of Inhibition, Mean ± sd)						
	*E. coli*	*S. infantis*	*P. fluorescens*	*B. cereus*	*S. aureus*	*L. monocytogenes*	*L. plantarum*
PS	18 ± 2	14 ± 2	22 ± 6	82 ± 3	76 ± 4	64 ± 12	1 ± 1
PSMME	54 ± 8	42 ± 20	50 ± 15	101 ± 6	76 ± 2	62 ± 15	0 ± 0
DHPSMME	15 ± 2	14 ± 8	7 ± 3	74 ± 6	30 ± 9	55 ± 16	4 ± 5

**Table 5:** MIC values of cicerfuran and its analogues from Aslam et al. (2009) [52].

Compound	MIC (µg/mL)	
	*B. subtilis*	*P. syringae*
1a	>400	>400
1b	>400	25
1c	>400	>400
1d	>400	>400
2a	>400	>400
2b	* nt	* nt
2c	>400	>400
2d	25	25
2e	25	50
3a	>400	>400
3b	>400	>400
3c	>400	>400
3d	>400	>400
3e	25	25
3f	>400	>400
** CAF	0.78	0.78

* nt = not tested; ** CAF= chloramphenicol, used as positive control

**Table 6:** MIC of some halogenated resveratrol derivatives from Li et al. (2012) [59].

Compound	MIC (µg/mL)	
	*E. coli* ATCC 25922	*S. aureus* ATCC 25923
Resveratrol	250	3.90
4a	31.3	31.3
4b	125	62.5
4c	62.5	31.3
4d	15.6	3.90
4e	31.3	7.81
* Levofloxacin	0.156	0.156

* Levofloxacin was used as positive control.

**Table 7:** Structural characteristics and antibacterial activity of the prenylated compounds from de Bruijn et al. (2018) [68].

Compound	Precursor	n° H-Bond Donors	LogD_7.2_	MIC (µg/mL)
Piceatannol	n.a.	n.a.	3.06	>200
Resveratrol	n.a.	n.a.	3.38	>200
Pinosylvin	n.a.	n.a.	3.69	≤100
Arachidin-1	Piceatannol	4	4.93	>50
Arachidin-2	Resveratrol	3	5.10	>50
Arachidin-3	Resveratrol	3	5.23	>50
Arachidin-6	Piceatannol	3	4.27	50–75
Arahypin-5	Resveratrol	2	4.58	25–50
Chiricanine A	Pinostilbene	2	5.40	12.5

n.a., not applicable. LogD_7.2_ calculated octanol-water partitioning coefficient at pH 7.2.

**Table 8:** Antibacterial activity of longistylin A (LLA) compared with vancomycin (VAN) from Wu et al. (2020) [69].

MIC [MBC] (µg/mL)					
Compound	MRSA JCSC 4744	MRSA JCSC 4469	*S. aureus* CMCC 26003	*B. cereus* CMCC 63302	*E. coli* ATCC 8739
LLA	1.56 [1.56]	1.56 [1.56]	1.56 [1.56]	25 [50]	>100 [>100]
VAN ^a^	0.78 [3.12]	0.78 [1.56]	1.56 [3.12]	50 [100]	50 [100]

MIC, minimum inhibitory concentration; in square brackets MBC, minimum bactericidal concentration; MRSA, methicillin-resistant *S. aureus*. ^a^ VAN (vancomycin), used as positive control.

**Table 9:** Antibacterial activity of hymenocardichrom-ene/anes against MRSA-108 S. aureus from Starks et al. (2014) [70].

Compound	MIC (µg/mL)
h. A	>32
h. B	16
h. C	16
h. D	16
h. E	16
h. F	16
h. acid	8
Vancomycin *	2

* Vancomycin was used as positive control.

**Table 10:** Antibacterial activity of stilbostemins, stemanthrene and compounds **8** and **9** in MIC values (µg/mL) from Yang et al. (2006) [71].

Compound	*S. aureus*	*S. epidermidis*	*E. coli*
S. J	>50	>50	>50
S. K	>50	>50	>50
S. L	50	12.5–25	>50
S. F	25	12.5–25	>50
8	12.5	12.5–25	>50
9	25	25–50	>50
Bakuchiol	25	12.5	50
Magnolol	25	12.5	50

Bakuchiol and Magnolol were used as positive control agents.

**Table 11:** Antimicrobial activity of compounds 10–13 in MIC values, µg/mL from Yang et al. (2016) [72].

Compound	*S. aureus*	*S. epidermidis*	*B. subtilis*	*E. coli*	*K. pneumoniae*
10	100	200	100	>200	>200
11	200	100	>200	>200	>200
12	200	200	200	>200	>200
b. B	6.25	25	50	200	>200
b. C	25	25	100	>200	>200
13	20	25–50	200	200	>200
Chloroamphenicol ^a^	4	4	8	2	

^a^ Chloroamphenicol was used as positive control.

**Table 12:** MIC of compounds isolated from African Combretaceae from Katerere et al. (2012) [73].

Compound	*E. coli*	*M. fortuitum*	*P. vulgaris*	*S. aureus*
14a	>100	25	100	25
14b	>100	25	100	25
14c	>100	100	100	100
15a	>100	25	100	25
15b	>100	25	100	25
16a	100	100	50	25
16b	>100	100	100	100
16c	>100	>100	>100	>100
16d	>100	>100	>100	>100
16e	>100	>100	>100	>100
Streptomycin *	3.12	1.56	3.12	0.78

* Streptomycin was used as positive control.

**Table 13:** Minimum inhibitory concentrations (MICs) for selected compounds isolated from Bletilla striata from Jiang et al. (2019) [74].

Compound	MIC (µg/mL)			
	*S. aureus* ATCC 6538	*B. subtilis* ATCC 6051	*MRSA S. aureus* ATCC 43300	*E. coli* ATCC 11775
18a	–	53	53	–
19a	26	53	53	–
19c	53	–	–	–
20	26	–	–	–
21	52	–	–	–
22	52	–	–	–
23	6	–	–	–
Oxacillin ^a^	0.078	1	3	137

^a^ Oxacillin was used as positive control.

**Table 14:** Antibacterial activity of the isolated compounds from Sahidin et al. (2017) [108].

Compound	Diameter of Inhibition Zone (Mean ±SD) *	
	*E. coli*	*S. aureus*
Balanocarpol	9 ± 0.17	13 ± 0.12
ɛ-Viniferin	11 ± 0.22	7 ± 0.17
α-Viniferin	8 ± 0.20	8 ± 0.16
Vaticanol B	5 ± 0.12	4 ± 0.14
Hopeaphenol	6 ± 0.16	8 ± 0.11
Tetracycline	14 ± 0.14	19 ± 0.12

* SD: standard deviation, triplicates; diameter of Whatman paper = 6 mm, (Balanocarpol) = (ɛ-Viniferin) = (α-Viniferin) = (Vaticanol B) = (Hopeaphenol) = 10,000 µg/mL; control (tetracyclin 30 µg/disc).

**Table 15:** MIC, MBC, and total bacterial adherence inhibition (TBAI) of the compounds from V. amurensis from Yim et al. (2010) [109].

Compound	*S. mutans*			*S. sanguis*		
	MIC (µg/mL)	MBC (µg/mL)	TBAI (µg/mL)	MIC (µg/mL)	MBC (µg/mL)	TBAI (µg/mL)
ɛ-Viniferin	25	50	25	12.5	50	50
(+)-Ampelopsin A	200	200	100	>400	>400	>400
(+)-Ampelopsin F	100	200	100	>400	>400	>400
Amurensin G	50	50	25	12.5	100	50
2-r-Viniferin	50	50	25	200	400	100
Erythromycin *	<1.5	<1.5	0.78	12.5	50	25

* Erythromycin was used as positive control.

**Table 16:** MIC values of δ-viniferin from Mora-Pale et al. (2015) [114].

Compound	MIC (µg/mL)				
	*B. cereus*	*L. monocytogenes*	*S. aureus*	*E. coli BL21*	*E. coli BL21 ΔTolC*
δ-Viniferin	13.6	113.5	28.1	> 113.5	3.2
δ-Viniferin + inhibitor	6.8	113.5	28.1	113.5	1.4

Efflux pump inhibitors: Phe-Arg-β-naphtylamide (PABN, 48.1 µM) for *E. coli*, 1(1-naphtylmethyl)-piperazine (NMP, 449.1 µM) for *B. cereus*, reserpine (16 µM) for *L. monocytogenes*, and piperine (350 µM) for *S. aureus*.

**Table 17:** MIC and MBC (in brackets) of resveratrol dimers against a panel of bacteria from Mattio et al. (2019) [36].

Bacteria	MIC (MBC) (µg/mL)			
	Viniferifuran	Dehydro-δ-viniferin	δ-Viniferin	Chlorexidine *
Gram-positive				
*L. monocytogenes Scott A*	16 (>512)	2(16)	16 (128)	8 (32)
*S. aureus* ATCC 25923	16 (>512)	2(16)	16 (512)	32 (128)
*E. faecium* DSM 20477	8 (>512)	2(32)	8 (512)	4 (128)
*E. faecalis* DSM 20478	8 (512)	4 (64)	16 (512)	8 (128)
*B. cereus* DSM 9378	4 (128)	1 (16)	4 (256)	8 (16)
Gram-negative				
*P. aeruginosa* ATCC 27853	128 (>512)	256 (512)	256 (>512)	32 (64)
*E. coli* DSM 682	256 (>512)	512 (>512)	256 (>512)	32 (64)
*E. coli* DSM 8579	256 (>512)	512 (>512)	256 (512)	32 (32)
*S. enterica* DSM 9386	256 (>512)	512 (>512)	256 (512)	32 (32)
*P. hauseri* DSM 30118	256 (>512)	512 (512)	256 (512)	32 (32)

* Chlorexidine was used as positive control.

**Table 18:** Results of checkerboard assays of colistin in combination with resveratrol from Cannatelli et al. (2018) [120].

Strains	Phenotype	Colistin MICs (µg/mL) at Different Resveratrol Concentrations (µg/mL)						
		Resveratrol Concentration (µg/mL)						
		0	8	16	32	64	128	256
*E. coli* LC711/14	COL-R	8	4	2 *	1 *	0.25 *	0.25 *	0.125 *
*E. coli* LC761/12	COL-R	4	0.5 *	0.5	0.25 *	0.125 *	0.125 *	0.125 *
*E. coli* FI-4451	COL-R	8	8	8	4	4	4	4
*E. coli* FI-4531	COL-R	8	4	4	4	4	2 *	2 *
*E. coli* FI-4592	COL-R	8	4	4	4	4	4	4
*E. coli* LC902/14	COL-R	8	4	4	4	4	2 *	2 *
*C. braakii* CA-26	COL-R	8	4	4	4	4	2 *	2 *
*S. maltophilia* 157	COL-R	32	4	2 *	1 *	0.5 *	0.125 *	0.125 *
*E. cloacae* CIP6085	COL-R	128	128	32 *	8 *	0.5 *	0.25 *	0.125 *
*K. pneumoniae* KKBO-1 ^a^	MDR/ COL-S	0.5	0.5	0.5	0.5	0.5 *	0.5	0.5
*K. pneumoniae* KKBO-4	MDR/ COL-R	64	64	64	16 *	8 *	4 *	4 *
*K. pneumoniae* KPB-1 ^a^	MDR/ COL-S	0.5	0.5	0.5	0.5	0.5	0.5	0.5
*K. pneumoniae* KPB-2	MDR/ COL-R	4	4	4	4	2	1 *	0.5 *
*K. pneumoniae* KK207-1 ^a^	MDR/COL-S	0.5	0.5	0.5	1	0.5	0.5	0.5
*K. pneumoniae* KK207-2	MDR/ COL-R	64	64	64	64	4 *	1 *	0.5 *
*K. pneumoniae* 6884	MDR/ COL-R	8	8	8	8	4 *	1 *	1 *
*K. pneumoniae* KPFan ^b^	COL-R	32	32	16	8 *	2 *	1 *	1 *
*K. pneumoniae* KPGP1 ^b^	COL-R	32	32	32	32	2 *	1 *	1 *
*A. Baumanii* N50	MDR/COL-R	64	64	64	64	8 *	1 *	1 *
*S. marcescens* CCUG1647^T^	COL-R	>128	>128	>128	>128	16 *	1 *	1 *
*P. mirabilis* NO-051	COL-R	>128	>128	>128	>128	>128	>128	8 *

^a^ These strains were the colistin-susceptible (COL-S) precursors of colistin-resistant (COL-R) *K. pneumoniae* KKBO-4, *K. pneumoniae* KPB-2, and *K. pneumoniae* KK207-2 strains, respectively. Resveratrol MICs were > 512 mg/L for all tested strains. ^b^ These COL-R strains were selected in vitro, using two COL-S precursors. Multidrug-resistant phenotypes (MDR) refer to strains resistant to carbapenems (imipenem and meropenem), ciprofloxacin, and amikacin. * Combinations in which colistin/resveratrol combinations yielded a synergistic activity (FICI ≤ 0.5). The lower concentration of resveratrol needed for restoring susceptibility to colistin is shaded in gray.

**Table 19:** MIC values of Res-AuNPs, Res-AgNPs-SDS, Res-AgNPs-NaOH, resveratrol standard, and norfloxacin, used as standard antibiotic from Park et al. (2016) [132].

Strains	MIC Values (µg/mL)				
	Res-AuNPs	Res-AgNPs-SDS	Res-AgNPs-NaOH	Resveratrol std	Norfloxacin
*S. aureus SG511*	>57	40	>80	114	< 0.25
*S. aureus 285*	>57	40	>80	>114	1
*S. aureus 503*	>57	40	>80	>114	1
*S. pyogenes 308°*	>57	80	80	57	8
*S. pyogenes T 12 A*	>57	80	>80	114	1
*S. pyogenes 77 A*	>57	80	>80	>114	4
*P. aeruginosa 9027*	>57	>80	>80	>114	1
*P. aeruginosa 1592E*	>57	>80	>80	>114	0.5
*P. aeruginosa 1771*	>57	>80	80	>114	0.5
*P. aeruginosa 1771 M*	>57	80	80	>114	0.25
*E. coli 078*	>57	>80	80	>114	<0.06
*E. coli TEM*	>57	>80	80	>114	0.12
*E. coli 1507*	>57	>80	>80	>114	<0.06
*E. coli DC 0*	>57	>80	80	>114	1
*E. coli DC 2*	>57	40	80	114	0.25
*S. typhimurium*	>57	80	>80	114	<0.06
*K. oxytoca 1082E*	>57	40	>80	>114	<0.06
*K. aerogenes 1522E*	>57	>80	>80	>114	0.12
*E. cloacae P99*	>57	>80	80	>114	<0.06
*E. cloacae 1321E*	>57	>80	80	>114	<0.06
*E. coli*	>57	>80	80	>114	<0.06
*S. pneumoniae*	14.25	40	>80	28.5	2
